# Mechanochemical Fullerene Nanoencapsulation in Amino‐Functionalized ZIF‐12 for Visible‐Light Disinfection of Waterborne Viruses and Bacteria

**DOI:** 10.1002/smll.202512881

**Published:** 2026-02-23

**Authors:** Noelia Rodríguez‐Sánchez, Menta Ballesteros, Carsten Prinz, Inés Canosa, Amando Flores Díaz, Biswajit Bhattacharya, A. Rabdel Ruiz‐Salvador, Franziska Emmerling

**Affiliations:** ^1^ Department of Materials Chemistry BAM Federal Institute For Materials Research and Testing Berlin Germany; ^2^ Departmento de Biología Molecular y Ingeniería Bioquímica Universidad Pablo de Olavide Sevilla Sevilla Spain; ^3^ Centro de Nanociencias y Tecnologías Sostenible Universidad Pablo de Olavide Sevilla Sevilla Spain; ^4^ Centro Andaluz de Biología del Desarrollo/Universidad Pablo De Olavide/Junta de Andalucía Sevilla Sevilla Spain; ^5^ Departmento de Sistemas Físicos, Químicos y Naturales Universidad Pablo de Olavide Sevilla Sevilla Spain

**Keywords:** fullerene, heterogeneous photo‐Fenton, mechanochemistry, nanoencapsulation, photocatalysis, ZIF‐12

## Abstract

Water contamination poses a significant threat to public health and environmental sustainability, necessitating the development of efficient purification technologies. This study reports the mechanochemical synthesis of an amino‐functionalized ZIF‐12 framework with encapsulated fullerene (C_60_) as a bifunctional photocatalyst for water decontamination and disinfection under visible‐light irradiation. The samples were characterized using powder X‐ray diffraction (PXRD), FT‐IR spectroscopy, N_2_ adsorption isotherm, X‐ray photoelectron spectroscopy (XPS), electron microscopy (SEM and TEM), thermogravimetric analysis (TGA), and UV–vis diffuse reflectance spectroscopy (DRS), confirming the retention of crystallinity and effective incorporation of C_60_ and amino groups within the framework. Fullerene loading and amino functionalization modified the optical properties, extending visible light absorption and enhancing charge separation and photocatalytic activity. The synergistic interaction between amino groups and C_60_ promotes efficient charge separation and enhanced hydroxyl radical production, resulting in improved photocatalytic and photo‐Fenton activity. C_60_@ZIF‐12‐NH_2_ exhibited excellent photocatalytic performance, achieving the complete inactivation of bacteriophage P22 under saline conditions and effective disinfection of *E. coli* and coliforms in natural river water, demonstrating robustness under environmentally relevant conditions. The solvent‐minimized mechanochemical synthesis and visible‐light‐driven activity position the C_60_@ZIF‐12‐NH_2_ composite as a promising platform for sustainable and advanced water treatment applications.

## Graphical abstract

A solvent‐minimized mechanochemical strategy enables the synthesis of C₆₀‐encapsulated amino‐functionalized ZIF‐12 as a visible‐light‐driven bifunctional photocatalyst. Synergistic charge separation enhances hydroxyl radical generation, achieving efficient viral in saline water and bacterial inactivation in river water under environmentally relevant conditions.

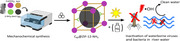

## 1 Introduction

The impact of nanotechnology‐based solutions for environmental remediation is continuously growing [1]. Particular attention has been centered on the development of materials and technologies to ensure universal access to clean water. Despite decades of scientific progress, clean water scarcity remains a defining challenge of our era, threatening environmental sustainability and global public health. Therefore, in recent years, attention has shifted not only to common pollutants but also to substances like pharmaceuticals [2], personal care products [3], pesticides [4], or microorganisms like waterborne viruses and bacteria [5], among others. These substances, originating from industrial, agricultural, and domestic activities [6], often persist through conventional treatments, posing significant risks to ecosystems and human health [7]. Recognizing this issue, regulatory frameworks such as European Directive 2024/3019 on urban wastewater treatment have introduced quaternary treatment processes to address the limitations of traditional methods, such as filtration and biological degradation, in effectively removing these pollutants [8].

In this context, engineering nanoporous materials has emerged as an attractive strategy to mitigate water pollution [9, 10], driven by the advantages associated with pore confinement in key processes such as adsorption, ion exchange, and catalysis [11]. For instance, zeolite nanopores have proven to be efficient hosts for capturing heavy metals, a property that has been largely explored for metal removal from water [12]. The elimination of emerging contaminants, however, requires materials with larger pore sizes, where pore confinement will be leveraged to enhance adsorption and catalytic performance. In this scenario, increasing attention has been progressively directed toward metal‐organic frameworks (MOFs) [13, 14, 15]. These materials have demonstrated excellent performance in water treatment applications for the removal of pharmaceuticals [16, 17], pesticides [16, 18], and per‐ and polyfluoroalkyl substances (PFAS) [19], among other emerging contaminants. Building on the experience gained with metal oxide semiconductors [20], MOFs exhibiting semiconducting properties are attracting growing interest for photocatalytic water treatment applications [14, 21, 22]. At the same time, a large amount of knowledge is being transferred from the field of Advanced Oxidation Processes (AOPs), which have emerged as a powerful strategy to address problems associated with water pollution. These processes rely on the generation of highly ROS, such as hydroxyl radicals (•OH), which can oxidize and mineralize contaminants into harmless by‐products and inactivate a wide range of microorganisms [23]. Among AOPs, heterogeneous Fenton‐like catalysis under visible light has gained particular attention due to its ability to effectively integrate successful advanced oxidation of a wide range of pollutants with sustainable light utilization [24, 25, 26], which has made it a technology that is already being applied on a pilot and semi‐industrial scale [27, 28]. Despite being an established technology, limitations must be overcome when the process uses iron salts in a homogeneous manner. The main limitation is the production of iron sludge during the neutralization step, which is necessary due to the acidic pH required. Therefore, interest in heterogeneous processes has increased in recent years. However, the efficacy of these processes depends critically on the development of robust photocatalysts that maintain high efficiency and structural integrity under operational and real conditions [29].

In this scenario, the application of MOFs in photo‐Fenton processes has recently been recognized as a promising approach for advanced water purification [30, 31]. MOFs employed in water treatment, particularly those relying on photo‐Fenton and other light‐driven technologies, require high structural and chemical stability. Consequently, most attention has focused on zeolitic imidazolate frameworks (ZIFs) [32, 33]. This is a subclass of MOFs, which has attracted considerable attention as advanced materials for environmental applications due to their exceptional chemical and thermal stability, high surface area, and tunable porosity [34, 35, 36], making them ideal candidates for adsorption, separation, and catalytic processes [37, 38, 39]. Moreover, their modular synthesis allows direct post‐synthetic functionalization and the formation of composites with other nanomaterials, such as fullerene, graphene oxides, or metal nanoparticles, to enhance reactivity, stability, or selectivity for target pollutants [40, 41, 42].

In a pioneering theoretical study, Grau‐Crespo and co‐workers demonstrated that the electronic structure of ZIFs can be systematically modulated by mixing both ligands and metals to fit the band gap in the optimal range for various applications, including photocatalysis [43]. Building on this foundation, our recent work reported the design of multivariate ZIFs exhibiting remarkable photocatalytic performance in both water decontamination and disinfection [44, 45]. Alternatively, to modify the framework composition at both metal and ligand sites, the electronic structure can be tuned by nanoencapsulating electro‐optical active species within the ZIFs nanopores. Among potential candidates, C_60_ stands out due to its versatility and outstanding performance in photocatalytic applications, where it acts as an electron buffer, enhancing both the activity and lifetime of the generated charge carriers [46, 47].

To encapsulate C_60_, ZIFs with free room larger than 11 Å are required. ZIF‐8 or ZIF‐67 could be suitable candidates, as shown by Martinez et al. [48]. However, due to the close match between the radius of C_60_ and the cage of these ZIFs, there is no additional space for other molecules to access the interior of the material. ZIF‐11 and ZIF‐12 are potential candidates, characterized by an RHO topology, benzimidazolate linkers, and a pore size of 14.6 Å. The generally higher photocatalytic activity of Co‐based MOFs compared to their Zn analogues makes ZIF‐12 the preferred option. In addition, its hydrophobic nature and relatively large pore size [49], offer promising potential for water remediation, particularly when combined with oxidative processes. Although ZIF‐12 has been investigated for adsorption [50], reports on its chemical functionalization are very limited and mostly restricted to post‐synthetic modifications with simple functional groups [51]. To date, no studies have systematically explored amino‐functionalization to introduce reactive sites and improve charge transfer. In a previous study, we demonstrated that substituting a small fraction of benzimidazole with a different imidazolate linker endows ZIF‐9 with enhanced catalytic properties [45]. We also noted that its hybridization with oxidative or photocatalytic components, such as fullerene‐based guests, has not yet been explored. Since the solvothermal synthesis has been identified as one of the main limitations in the application of MOFs for water treatment [52], greener routes are desired. Likewise, solvent‐free mechanochemical routes remain essentially unexplored for ZIF‐12. As a more sustainable and scalable alternative, mechanochemical synthesis involves the grinding of solid precursors in the absence or near absence of solvents, offering a green, energy‐efficient, and scalable route to produce advanced materials [53, 54]. Compared to conventional solvothermal methods, mechanochemistry eliminates the need for large volumes of solvents and reduces reaction times, making it an environmentally friendly alternative [53, 55, 56]. Shinde et al., demonstrated that MOFs can be mechanochemically activated, showing high potential as a filler in polymer networks for mechanochemical sensing of friction and wear in materials such as polyurethanes or epoxies [30]. Mechanochemical synthesis also facilitates the incorporation of functional linkers and guest molecules, enabling the synthesis of complex composites with enhanced properties. Brekalo et al. demonstrated that mechanochemical synthesis of a bare imidazole RHO topology requires a template [57], in contrast to ZNI, ZIF‐4 (CAG) and ZIF‐6 (GIS) topologies [58], which reflects the well‐known inverse relation between stability and density in ZIFs [59, 60]. Theoretical calculations by Lewis et al. also showed that functionalized imidazolate ligands can partially serve a templating role [59]. Using 2‐ethylimidazole under mechanochemical conditions, Beldon et al. prepared an RHO ZIF, exhibiting very low stability [61]. In contrast, employing benzimidazole in conjunction with toluene as mixing templating agents, ZIF‐11 and ZIF‐12 can be prepared at room temperature, while the absence of toluene leads the synthesis to the less porous SOD‐topology ZIF‐7 and ZIF‐9 solids [49]. In this context, we hypothesize that C_60_ could act as a co‐template agent for the mechanochemical synthesis of ZIF‐12, with the additional advantage that C_60_ remains encapsulated and available for photochemical applications. Herein, we report, for the first time, the mechanochemical synthesis of ZIF‐12 and amino‐functionalized ZIF‐12, together with the nanoencapsulation of fullerene (C_60_) within this framework to obtain a bifunctional, visible‐light‐active photocatalyst. In contrast to previous studies, where either framework functionalization or carbon‐based additives were explored separately, this work demonstrates that the combined incorporation of amino groups and C_60_ within a single ZIF‐12 framework leads to a synergistic enhancement of charge separation and reactive oxygen species generation, resulting in improved photocatalytic and photo‐Fenton activity. The resulting functional material, C_60_@ZIF‐12‐NH_2_, combines the hydrophobicity and porosity of ZIF‐12 with the photocatalytic properties of fullerenes and the increased reactivity afforded by amino‐functionalization. As proof of concept, we investigated the removal of a model contaminant, MB, under visible‐light photocatalysis and heterogeneous Fenton‐like catalysis. Moreover, this study provides the first demonstration of bacteriophage P22 inactivation using a ZIF‐based photocatalysis and validates its effectiveness for both disinfection and decontamination of water, including the disinfection of natural water matrices (river water) under realistic conditions, where natural organic matter and dissolved ions may interfere, thereby bridging the gap between fundamental material design and practical water applications. Finally, the evaluation of material stability and recyclability was performed to ensure the reuse of the catalyst over several consecutive cycles.

## 2 Results and Discussion

### 2.1 Characterization

**FIGURE 1:**
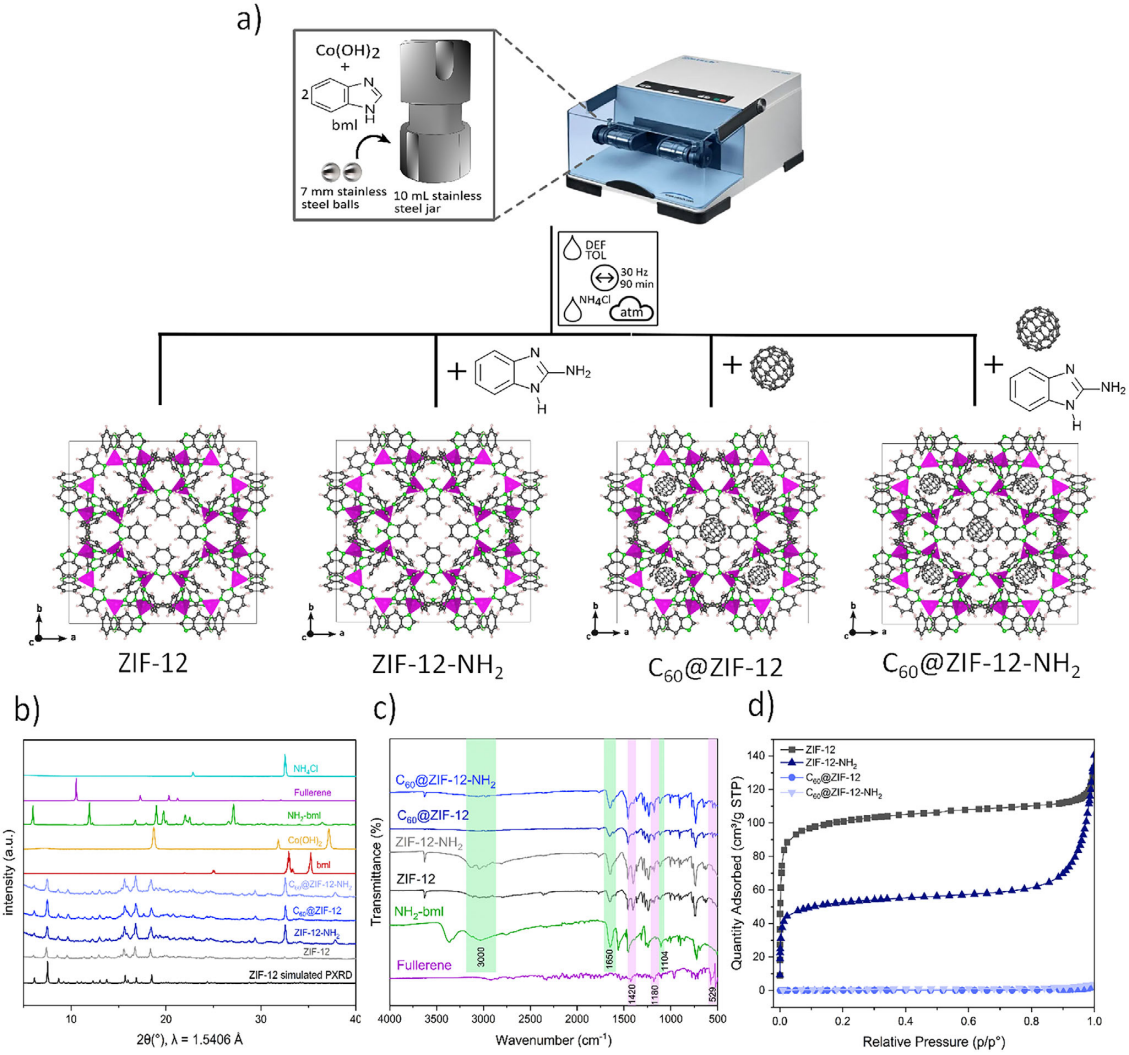
Schematic illustration of the mechanochemical synthesis of ZIF‐12‐NH_2_, C_60_@ZIF‐12, and C_60_@ZIF‐12‐NH_2_ (a), PXRD diffractogram (b), FT‐IR spectra (c), and N_2_ adsorption isotherm of ZIF‐12, ZIF‐12‐NH_2_, C_60_@ZIF‐12, and C_60_@ZIF‐12‐NH_2_ (d).

Figure 1a shows a schematic view of the synthesis of ZIF‐12 and its amino‐functionalized variant (ZIF‐12‐NH_2_), as well as the one‐pot synthesis of both frameworks with encapsulated C_60_ fullerenes within the ZIF cages to form C_60_@ZIF‐12 and C_60_@ZIF‐12‐NH_2_ composites. All characterizations were performed on the samples containing 12 mg (4%) of C_60_, which were identified as the optimal loading based on the photocatalytic studies. The selection of the initial parameters for the mechanochemical synthesis of the materials was based on our previous optimization studies carried out for the synthesis of ZIF‐9, which corresponds to the SOD topology of ZIF‐12 [62]. Given the structural similarity between ZIF‐9 and ZIF‐12, we used these conditions as a starting point and subsequently optimized the parameters to achieve the best results for this material. PXRD was employed to investigate the crystallinity of the materials (Figure 1b). The characteristic diffraction peaks of ZIF‐12 were observed at 2θ values of 6° (002), 7° (112), and 8° (022), confirming the successful first‐time mechanochemical synthesis of ZIF‐12. The high intensity of these peaks indicates a high degree of crystallinity in the synthesized ZIFs, which is consistent with previous studies of ZIF‐12 prepared by wet chemistry [49, 63]. Upon the incorporation of 2‐NH_2_‐bml, the diffraction pattern of ZIF‐12‐NH_2_ shows no significant shift in peak positions, indicating that the structural integrity of the ZIF‐12 framework is maintained. However, slight variations in relative peak intensities suggest possible changes in preferred crystallite orientation or minor distortions due to ligand functionalization [64]. The characteristic peaks of C_60_@ZIF‐12 and C_60_@ZIF‐12‐NH_2_ at 6° (002), 7° (112), and 8° (022) remain largely consistent with those of their respective parent frameworks, demonstrating that the overall structure remains intact after fullerene loading. The absence of significant peak broadening suggests that the encapsulation process does not induce amorphization, and the crystallinity of the host frameworks is preserved. Furthermore, the absence of Bragg peaks corresponding to the reagents indicates complete incorporation of the precursor materials into the MOF framework [65].

The ZIF‐12 FT‐IR spectrum displays characteristic vibrational bands associated with the benzimidazolate linker (Figure 1c). The most prominent peaks include C─C stretching (∼1460 cm^−^^1^) and the bands identified in the region of 1500–1350 cm^−1^ assigned to various stretching vibrations of the imidazole ring, which are consistent with the literature‐reported spectra of ZIF‐12 [63]. Upon functionalization, the spectrum of ZIF‐12‐NH_2_ and C_60_@ZIF‐12‐NH_2_ exhibits higher intensities at ∼1650 cm^−^^1^,∼3000 cm^−^^1^, and 1104 cm^−^^1^, and belonging to N─H and C─N bonds from amine groups, respectively [66, 67]. This functionalization does not significantly alter the ZIF‐12 bands, reinforcing the diffraction results that the framework remains structurally intact. Minor shifts in the C─N stretching frequencies from 1103 to 1012 cm^−^^1^ indicate slight electronic modifications due to the presence of the amino groups, which may influence the electronic structure and reactivity of the material. Furthermore, the encapsulation of C_60_ within C_60_@ZIF‐12 and C_60_@ZIF‐12‐NH_2_ leads to additional spectral changes. The characteristic C_60_ vibrational bands appear at ∼1180 cm^−^^1^ (C═C stretching) and ∼529 cm^−^^1^ (C─C stretching) attributed to a tangential motion of carbon atoms and to radial motion of its carbon atoms, respectively [48], which are consistent with the fingerprint region of fullerene. Additionally, the band at 750 cm^−1^ attributed to the in‐plane imidazole ring deformation remains unchanged, while out‐of‐plane imidazole ring deformation at 760 cm^−1^ slightly increases its intensity, in concordance with a previous study of encapsulated C_60_ in ZIF‐8 [48]. When comparing the progressive encapsulation of C_60_ loading in C_60_@ZIF‐12 (Figure S1), distinct changes can be observed in the spectral profile. A gradual increase in intensity in the region of 700 cm^−1^ corresponding to the typical vibrational modes of C_60_, which become more prominent with higher fullerene content. Simultaneously, C_60_@ZIF‐12 and C_60_@ZIF‐12‐NH_2_ bands, especially in the fingerprint region, below 1500 cm^−^^1^, exhibit slight shifts and broadening, suggesting interaction between the C_60_ molecules and the framework [67]. Additionally, notable reductions in the intensity of the C_60_@ZIF‐12‐NH_2_ imidazolate vibrations around 1580–1640 cm^−^^1^ and 1140 cm^−^^1^ were noted with increasing C_60_, indicative of partial pore occupation or host‐guest interactions [35].

The nitrogen adsorption–desorption isotherms of ZIF‐12 reveal a type I isotherm with an adsorption capacity of approximately 140 cm^3^ g^−^^1^ and a BET surface area of 391.77 m^2^/g, indicative of a predominantly microporous structure (Figure 1d; Table S1). This is consistent with previously reported values for ZIF‐12 prepared by different synthetic routes. The literature reports a wide dispersion of BET surface areas for ZIF‐12, ranging from 24.92 m^2^ g^−^^1^ to values above 1100 m^2^ g^−^^1^, reflecting differences in synthesis methodology, crystallinity, particle size, defect density, and activation procedures [50, 51, 68, 69, 70]. The addition of amino groups in ZIF‐12‐NH_2_ leads to a similar profile with a final adsorption capacity of 140 cm^3^ g^−^^1^ and a BET surface area of 200.20 m^2^/g. Despite both ZIF‐12 and ZIF‐12‐NH_2_ reaching a similar total nitrogen adsorption at high relative pressures, ZIF‐12 exhibits a higher nitrogen adsorption in the microporous region at lower relative pressures, reaching 100 cm^3^ g^−^^1^, whereas ZIF‐12‐NH_2_ reaches 50 cm^3^ g^−^^1^ and a notably lower BET surface area, highlighting the partial pore blocking or restricted diffusion introduced by amino functionalization [71, 72, 73]. In contrast, both C_60_‐encapsulated materials, C_60_@ZIF‐12 and C_60_@ZIF‐12‐NH_2_, also exhibit a type I isotherms with a drastic decrease in the adsorption capacity and BET surface area of 1.29 and 2.55 m^2^/g for C_60_@ZIF‐12 and C_60_@ZIF‐12‐NH_2_, respectively. This behavior points to significant pore blocking and reduced accessibility of the internal surface area, indicating a non‐porous nature likely resulting from the fullerene nanoencapsulation [74, 75].

XPS was employed to analyze the surface chemical composition and electronic environment of the ZIF‐12‐based materials. The survey spectra confirmed the presence of the expected elements (C, N, O, Co, and Cl) in all samples (Figure S2). A component at 400.5 eV in the N 1s region (Figure S3) attributable to amino nitrogen was identified in all the samples due to the presence of NH_4_Cl, which is predominantly located on external surfaces remaining in the structure after synthesis, a typical behavior for ammonium salts in mechanochemical synthesis, in concordance with PXRD [76]. However, a significant increase of this peak rises from 18.76% in ZIF‐12 to 25.95% and 24.62% in ZIF‐12‐NH_2_ and C_60_@ZIF‐12‐NH_2_, respectively (Figure S2a,b,d). This increase, accompanied by a decrease in the original amine nitrogen (from 30.19% to 17.04% and 19.05% in ZIF‐12‐NH_2_ and C_60_@ZIF‐12‐NH_2_, respectively), confirms the partial replacement of the original ligand [77]. This interpretation is consistent with the shifts and area changes observed in XPS studies of amino‐modified framework materials, where amine‐related components increase at the expense of amine/other nitrogen species upon functionalization [12, 13]. C─C/C─H peaks were identified in the C 1S region in all the samples, fitting with the expected components of the ZIF‐12 framework (Figure S4). The functionalization further induces detectable electronic changes in the C 1s region (Figure S4b), where a π–π* shake‐up peak appears at 292.3 eV exclusively in ZIF‐12‐NH_2_, indicative of increased conjugation in the aromatic system due to the incorporation of the electron‐donating amino group [78, 79]. This satellite is absent in C_60_@ZIF‐12‐NH_2_ (Figure S4d), indicating a substantive electronic interaction between the functionalized framework and the fullerene, suggesting ground‐state charge transfer or electronic coupling, which modifies the local density of states of the aromatic linkers.

The Co 2p region confirmed the stability of the cobalt oxidation state across all materials (Figure S5). The characteristic spectral features, including main Co 2p_3/2_ peaks between 780.3 and 783.6 eV and intense shake‐up satellites at higher binding energies (∼786–806 eV), are assigned to high‐spin Co^2^^+^ in a tetrahedral coordination environment, consistent with the ZIF structure (Figure S5a) [80, 81]. Quantitative analysis of the peak areas revealed subtle electronic modifications upon functionalization and hybridization (Figure S5b–d). The relative area of the main Co 2p_3/2_ component at 781.6 eV increased from 21.29% in ZIF‐12 to 23.17% in ZIF‐12‐NH_2_ and further to 25.39% in C_60_@ZIF‐12‐NH_2_. Concurrently, a decrease in the relative intensity of the associated satellite peak is observed. Changes in the satellite‐to‐main peak intensity ratio in Co 2p XPS spectra have been widely interpreted as redistributions of electron density around cobalt centers induced by electronic interactions with functionalized ligands rather than by changes in formal oxidation state [82, 83].

TGA analysis was employed to evaluate the thermal stability and compositional changes of ZIF‐12, ZIF‐12‐NH_2_, C_60_@ZIF‐12 and C_60_@ZIF‐12‐NH_2_ (Figure S6). ZIF‐12 exhibits a gradual weight loss up to 250°C, attributed to the removal of solvent molecules, followed by a pronounced decomposition step between 500°C and 600°C, corresponding to the breakdown of the organic linkers. The residue percentage at 900°C is approximately 40 wt%, in agreement with previous studies [80, 84]. ZIF‐12‐NH_2_ and C_60_@ZIF‐12 exhibit a similar thermal profile, though with a slightly earlier step of decomposition between 250 and 400°C and a residue percentage of 35 and 20 wt%, respectively. Interestingly, C_60_@ZIF‐12‐NH_2_ reveals a similar weight loss step to ZIF‐12, differing from ZIF‐12‐NH_2_ and C_60_@ZIF‐12 in the initial loss of weight, confirming the stabilizing interaction between amino groups, fullerene, and the framework proposed at evaluating the FTIR data (Figure 1b).

**FIGURE 2:**
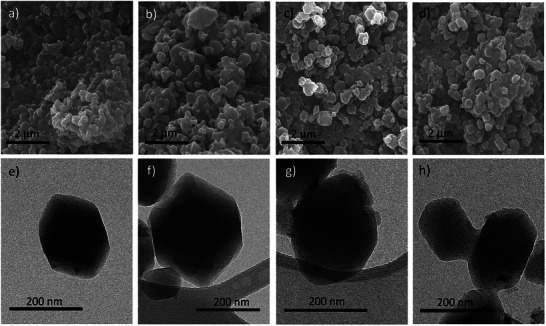
SEM images of (a) ZIF‐12, (b) ZIF‐12‐NH_2_, (c) C_60_@ZIF‐12, and (d) C_60_@ZIF‐12‐NH_2_, TEM images of (e) ZIF‐12, (f) ZIF‐12‐NH_2_, (g) C_60_@ZIF‐12, and (h) C_60_@ZIF‐12‐NH_2_.

The morphological and additional structural characterization of the synthesized materials was conducted using scanning electron microscopy (SEM) and transmission electron microscopy (TEM) (Figure 2a–f). The SEM images reveal distinct morphological differences between ZIF‐12, C_60_@ZIF‐12, ZIF‐12‐NH_2_, and C_60_@ZIF‐12‐NH_2_. SEM images of ZIF‐12 (Figure 2a) reveal polyhedral crystals with smooth surfaces that are less well defined than those reported in ZIF‐12 prepared by the solvothermal method [49]. This is probably due to the mechanical forces applied during the mechanochemical synthesis. The introduction of the amino group further influences the particle morphology, as observed in ZIF‐12‐NH_2_ (Figure 2b), where the crystal facets appear slightly less defined compared to ZIF‐12, indicating potential modifications in the growth kinetics during synthesis caused by the expected random distribution of the minority NH_2_‐imidazole ligand. When C_60_ is incorporated into C_60_@ZIF‐12 (Figure 2c), the resulting ZIF exhibits slightly rougher particle surfaces and minor aggregation as compared to ZIF‐12 but better than ZIF‐12‐NH_2_. This suggests that incorporating C_60_ into ZIF‐12 cages potentially alters the surface interactions without significantly disrupting the overall crystal morphology. However, in the case of C_60_@ZIF‐12‐NH_2_ (Figure 2d), a morphology closely resembling that of the non‐functionalized C_60_@ZIF‐12 is observed, albeit with a tendency toward greater particle agglomeration.

TEM images provide further insights into the encapsulation of C_60_ and the structural integrity of the materials at the nanoscale. The ZIF‐12 (Figure 2e) particles exhibit well‐defined hexagonal shapes with sharp edges and average particle size of approximately 200 nm, reaffirming their high crystallinity, while ZIF‐12‐NH_2_ (Figure 2f) maintains a comparable morphology with slightly smoother edges and bigger size of approximately 300 nm, suggesting an influence of the functionalization process on the crystallization pathway [73]. Upon fullerene incorporation, ZIF‐12–C_60_ retains a comparable polyhedral morphology with a slightly increased average particle size of around 250 nm. The amino‐functionalised composite (C_60_@ZIF‐12‐NH_2_) also preserves the characteristic ZIF‐12 morphology, displaying slightly smoother edges and an average particle size of approximately 200 nm. Upon fullerene incorporation, C_60_@ZIF‐12 and C_60_@ZIF‐12‐NH_2_ (Figure 2g,h) retains a comparable polyhedral morphology with a slightly increased average particle size of around 250 nm. The presence of fullerene in both materials is clear from the contrast variations observed in the TEM images, where a less uniform internal structure is observed that could be connected to the presence of interacting guest molecules with the framework [85]. High‐resolution TEM (HRTEM) images further confirm the retention of the crystalline nature of the materials (Figure S7), Notably, the encapsulation of C_60_ does not induce to amorphisation but rather introduces localized changes in contrast and texture.

**FIGURE 3:**
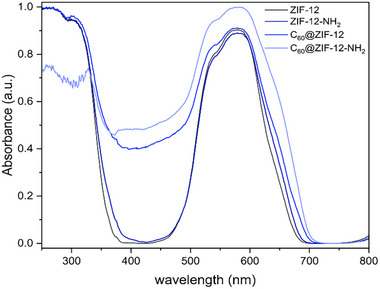
DRS spectra of ZIF‐12, ZIF‐12‐NH_2_, C_60_@ZIF‐12, and C_60_@ZIF‐12‐NH_2._

DRS spectra provide insight into the optical properties of the synthesized materials (Figure 3). ZIF‐12 exhibits a characteristic reflectance profile with a fundamental absorption in the UV region and a strong absorbance in the visible region, in the range of 500–650 nm, indicative of d‐d transitions [86], as has been previously reported for ZIF‐12 [87]. Upon functionalization with the NH_2_ group, a shift in the absorption range, suggests a slight reduction in the band gap due to the introduction of electron‐donating groups, which can facilitate charge transfer [88]. This occurs because the lone electron pair from NH_2_ increases the surface electron density near the imidazole ring of ZIF‐12, resulting in a shielding effect that reduces the binding energy [89]. The incorporation of C_60_ into ZIF‐12 and ZIF‐12‐NH_2_ leads to further modifications in the optical response, particularly a noticeable broadening of the DRS spectrum, extending further into the UV and visible region from 320 to 670 nm. This enhancement in light absorption can be attributed to the strong π‐π interactions between the ZIF framework and C_60_, which may facilitate improved charge separation and transfer [78, 79]. Additionally, C_60_@ZIF‐12‐NH_2_ reveals a slight absorption decrease in the UV range and extends the light absorption in the same range of 350–700 nm, suggesting strong host–guest interactions and possible electronic coupling between the encapsulated C_60_ and the ZIF‐12‐NH_2_ framework [90, 91, 92], potentially altering its electronic structure and improving its potential for photocatalytic applications [91].

### 2.2 Photocatalytic Activity of Materials

#### 2.2.1 Effect of Fullerene Loading

**FIGURE 4:**
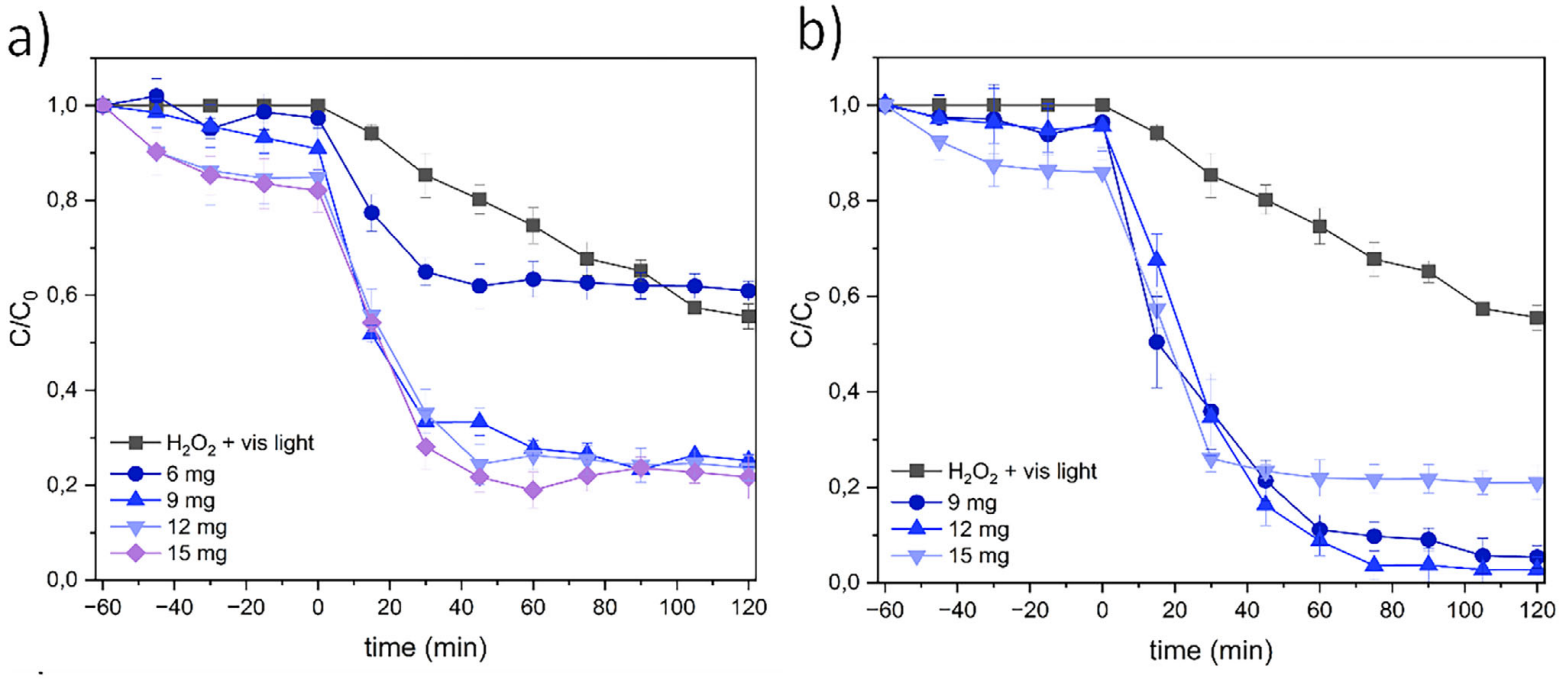
Degradation of (a) MB using C_60_@ZIF‐12 with different C_60_ loading by heterogeneous photo‐Fenton and (b) using C_60_@ZIF‐12‐NH_2_ with different C_60_ loading by heterogeneous photo‐Fenton.

The influence of fullerene content on ZIF‐12 photo‐Fenton activity was evaluated by incorporating 6 (∼2%), 9 (∼3%), 12 (∼4%), and 15 mg (∼5%) of C_60_ (Figure 4a) and using MB as a model contaminant. First, dye adsorption studies performed in the dark were carried out at the beginning of all the decontamination experiments (Figure 4a). Approximately 5% of MB for 2% of C_60_, 10% of MB for 3% mg of C_60_ and 15% of MB for both 4% and 5% of C_60_ were adsorbed after 60 min in dark. This increase in the adsorption can be explained due to the C_60_‐MB interactions, as has been reported in a aqueous adsorption study [93]. After turning on the lights, the photo‐Fenton results show that 2% (6 mg) of fullerene did not produce any significative improvement in MB degradation compared with the effect of H_2_O_2_ and light without a catalyst. However, when the fullerene content increased to 3% (9 mg), the degradation efficiency improved, reaching 80% of MB degradation after 120 min. Beyond this threshold, higher loadings produced no further improvement. Kinetic analysis revealed a transition from pseudo‐second‐order behavior at 6 mg (adsorption‐controlled) to pseudo‐first‐order kinetics at ≥9 mg (Table S2), indicating that once a sufficient amount of catalyst is available, the process becomes primarily governed by pollutant concentration [94]. (Table S2). The decontamination results for C_60_@ZIF‐12‐NH_2_ are shown in Figure 4b. Adsorption was found to be similar in all cases, with a slight increase in adsorption of MB with the increase of the amount of C_60_. Notably, the best photocatalytic performance was achieved with 9–12 mg C_60_, reaching 97% MB degradation after 120 min, while 15 mg resulted in decreased efficiency (80%). This shift from pseudo‐first‐order to pseudo‐second‐order behavior (Table S3) can be attributed to the saturation effect of fullerene in the framework, blocking the active sites or hindering the accessibility of ROS, as well as a recombination effect [95, 96]. These results suggests that an optimal balance between ZIF‐hosted active sites and C_60_‐mediated charge transfer is necessary for maximizing efficiency [97, 98]. Therefore, a C_60_ loading of 12 mg (4%) was selected for subsequent experiments.

#### 2.2.2 Synergistic Effect of Amino Functionalization and Fullerene Encapsulation

**FIGURE 5:**
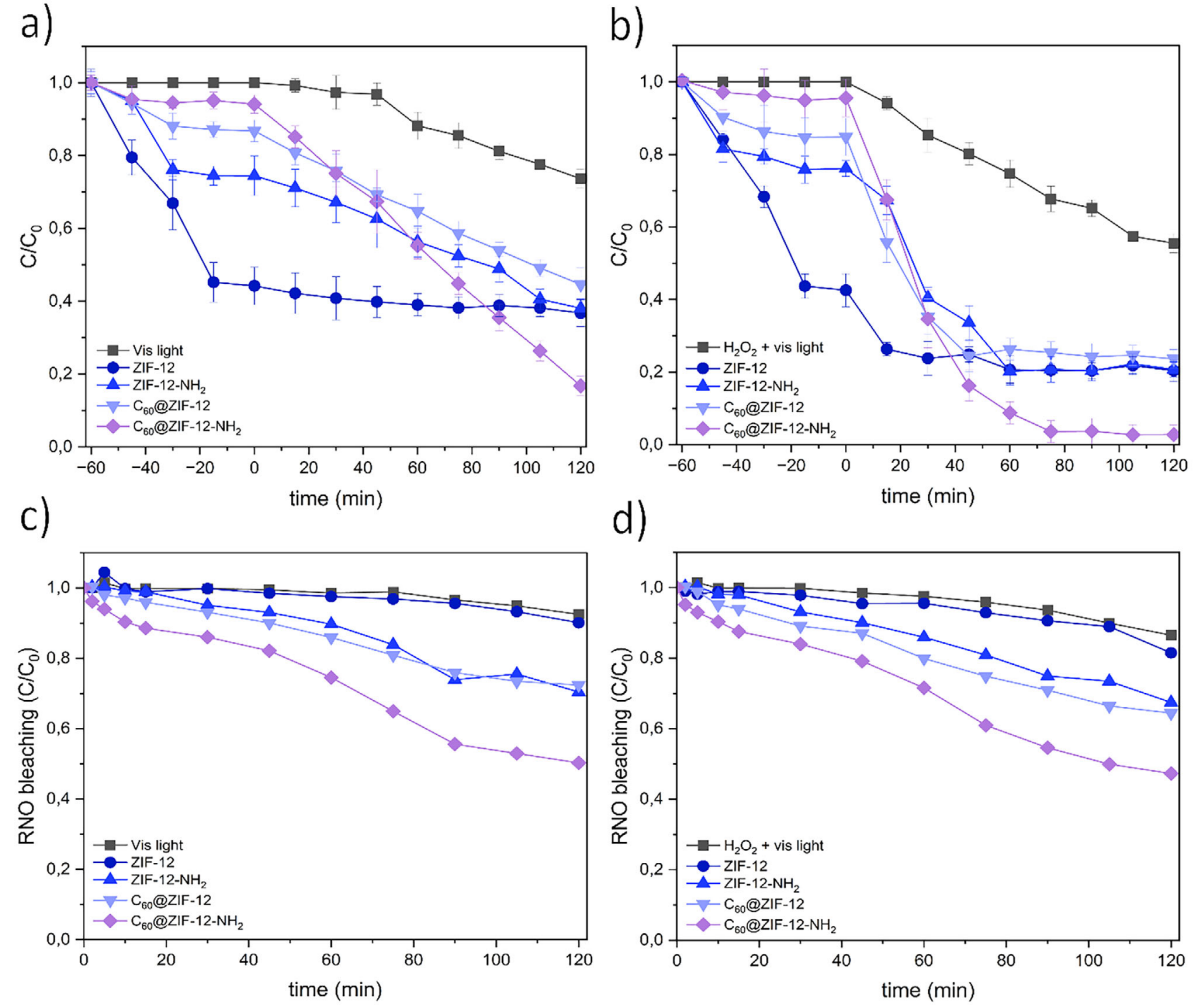
Degradation of MB with (a) ZIF‐12, ZIF‐12‐NH_2_, C_60_@ZIF‐12, and C_60_@ZIF‐12‐NH_2_ as catalyst by photocatalysis and with (b) ZIF‐12, ZIF‐12‐NH_2_, C_60_@ZIF‐12, and C_60_@ZIF‐12‐NH_2_ through heterogeneous photo‐Fenton and RNO bleaching using with (c) ZIF‐12, ZIF‐12‐NH_2_, C_60_@ZIF‐12, and C_60_@ZIF‐12‐NH_2_ under photocatalysis and (d) heterogeneous photo‐Fenton. MB concentration = 0.005 g/L, catalyst concentration = 0.05 g/L (C_60_ content12 mg for C_60_@ZIF‐12 and C_60_@ZIF‐12‐NH_2_), H_2_O_2_ concentration = 10 mm (without H_2_O_2_ for photocatalysis assays), irradiance = 1032 W/cm^2^.

A direct comparison between the photochemical behavior of ZIF‐12, ZIF‐12‐NH_2_, C_60_@ZIF‐12, and C_60_@ZIF‐12‐NH_2_ under visible‐light irradiation clearly demonstrates the positive synergistic effect of C_60_ encapsulation and functionalization (Figure 5a,b). The comparison includes photochemical experiments without (referred to as photocatalysis) and with H_2_O_2_ (referred to as photo‐Fenton). First, in dark conditions, adsorption was approximately 60% of MB for ZIF‐12, while 25%, 15%, and 10% of MB were adsorbed for ZIF‐12‐NH_2_, C_60_@ZIF‐12, and C_60_@ZIF‐12‐NH_2_, respectively. These results are consistent with the successful incremental incorporation of C_60_ in ZIF‐12 frameworks and the consequent partial pore blocking for the MB adsorption. The adsorption kinetics are strongly influenced by structural modification (Figure S8 and Table S4). While ZIF‐12 exhibits the highest adsorption capacity (50.8 mg g^−^^1^) and intraparticle diffusion rate (k_id_ = 0.492 mg g^−^^1^ min^−1/2^), in line with its high porosity and surface area, ZIF‐12‐NH_2_ leads to a pronounced decrease in adsorption capacity (3.40 mg g^−^^1^) despite relatively fast kinetics (k_1_ = 0.071 min^−^^1^), indicating that reduced porosity limits overall uptake while chemisorption at amino sites remains dominant. C_60_ incorporation further suppresses adsorption (2.29 mg g^−^^1^) and diffusion (k_id_ = 0.254 mg g^−^^1^ min^−1/2^) due to pore occupation, with adsorption behavior better described by a physical diffusion‐controlled mechanism. The combined modification in C_60_@ZIF‐12‐NH_2_ results in the lowest adsorption capacity (0.73 mg g^−^^1^) and slowest kinetics (k_1_ = 0.0333 min^−^^1^), reflecting the cumulative effects of pore blocking and framework alteration, in concordance with N_2_ adsorption results.

After the lights were turned on during the intrinsic photocatalysis tests (Figure 5a), ZIF‐12 exhibited negligible photocatalytic activity. The incorporation of fullerene and amino groups in C_60_@ZIF‐12 and ZIF‐12‐NH_2_ led to a slight increase in the photocatalytic performance, achieving a 50% degradation of MB after 120 min. In contrast, the bifunctional composite, C_60_@ZIF‐12‐NH_2_, exhibited a markedly enhanced performance, achieving ∼80% degradation within the same time frame. The degradation of MB under photocatalysis conditions followed pseudo‐first‐order kinetics, with the rate constant of C_60_@ZIF‐12‐NH_2_ being approximately double that of ZIF‐12‐NH_2_ and ∼1.6 times higher than that of C_60_@ZIF‐12 (Table S5). The efficiency of these materials was also tested by employing H_2_O_2_ to carry out the degradation of the model molecule by heterogeneous photo‐Fenton (Figure 5b). ZIF‐12 exhibited a slow efficiency under light irradiation, degrading only 20% of MB, likely due to the ROS generated by the combination of light irradiation and H_2_O_2_, as it was previously confirmed (Figure 5b). Similarly to the photocatalysis tests in the absence of H_2_O_2_, C_60_@ZIF‐12 and ZIF‐12‐NH_2_ exhibit significantly enhanced photo‐Fenton catalytic activity, reaching 80% MB degradation. However, the degradation process does not continue beyond 60 min, likely due to a possible deactivation of catalytic sites, depletion of H_2_O_2_, or recombination effects limiting further degradation [96, 97], while C_60_@ZIF‐12‐NH_2_ achieves the degradation of 90% of MB within 60 min (Figure 5b).

The degradation of MB fitted well to pseudo‐first‐order kinetics for ZIF‐12, C_60_@ZIF‐12, and C_60_@ZIF‐12‐NH_2_, with the rate constant increasing more than 600‐fold when going from ZIF‐12 to the bifunctional composite (Table S6). Conversely, ZIF‐12‐NH_2_ followed a pseudo‐second‐order model. Other studies reported the synthesis of CdS‐C_60_/TiO_2_ composite nanomaterials whose photocatalytic efficiency under visible light was CdS‐C_60_/TiO_2_> CdSC_60_> CdS‐TiO_2_. Similar to our results, the CdS‐C_60_/TiO_2_ composite exhibited the characteristics of both C_60_ and CdS, demonstrating the highest photocatalytic activity [99]. It is noteworthy that although ZIF‐12‐NH_2_ possesses amino groups that enhance MB adsorption under both conditions, the kinetic behavior differs between photocatalysis and photo‐Fenton. Under photocatalysis, the process is mainly controlled by ROS generation on the catalyst surface, leading to a pseudo‐first‐order kinetic profile. In contrast, in photo‐Fenton conditions, the simultaneous interaction of MB and H_2_O_2_ at the catalyst surface results in a dual dependence on both contaminant concentration and oxidant activation. This adsorption–reaction interplay is more accurately described by a pseudo‐second‐order kinetic model [100, 101]. Similar kinetic behavior has been reported for NH_2_‐MIL‐88B(Fe) or MOF‐235, among others [101, 102]. Despite the low adsorption performance, C_60_@ZIF‐12‐NH_2_ exhibits the fastest photocatalytic degradation under irradiation, demonstrating that high adsorption capacity is not a prerequisite for efficient photocatalysis. Weak and reversible adsorption facilitates rapid substrate turnover and prevents active‐site blocking, highlighting a trade‐off between adsorption and photocatalytic efficiency that is critical for material design. The weak, reversible adsorption observed in C_60_@ZIF‐12‐NH_2_ likely facilitates photocatalytic degradation by (i) preventing strong, irreversible MB binding that would deactivate catalytic sites, (ii) maintaining MB molecules in solution where they are more accessible to photogenerated reactive species, and (iii) enabling rapid substrate turnover at the photocatalyst surface [103]. These results are in concordance with several studies [104, 105, 106, 107]. The dual modification strategy creates a favorable interfacial environment for photocatalytic processes, potentially through enhanced charge separation, modified band structure, and improved surface reactivity [103].

It is well established that there is a clear correlation between band gap energies of the materials and their photocatalytic efficiency. Using Tauc plots, the band gaps obtained for ZIF‐12 and ZIF‐12‐NH_2_ were 3.41 and 3.09 eV, respectively. The decrease in band gap observed for ZIF‐12‐NH_2_ corresponds to improvement in photocatalytic activity and shifts the light absorption to the visible region, as the introduction of amino groups facilitates enhanced electron‐donating properties, promoting more efficient charge separation and •OH generation [108]. Notably, the incorporation of C_60_ significantly reduced the band gap values to 2.72 eV for C_60_@ZIF‐12 and 2.81 eV for C_60_@ZIF‐12‐NH_2._ This band gap narrowing is likely due to the electron‐accepting nature of C_60_, which enhances charge separation and extends the light absorption range into the visible spectrum [109]. Interestingly, C_60_@ZIF‐12 with a narrower light absorption spectrum, results in slower MB degradation kinetics compared to C_60_@ZIF‐12‐NH_2_. This can be explained by the electron‐acceptor nature of C_60_, which can facilitate charge separation, but if the transfer to reactive sites is hindered or recombination occurs too quickly, the catalytic activity may not be sustained [46]. However, the combination of C_60_ molecules and amino groups leads to strong π–π interactions between the C_60_ molecules and the ZIF framework, which enhance light absorption and improve charge separation [109]. While amino groups facilitate ligand‐to‐metal charge transfer transitions, the lone electron pair on the amine molecules participates in extended conjugation throughout the ZIF structure, improving charge separation and reducing recombination rates during the photo‐Fenton‐like process [108, 110, 111]. Consequently, the synergistic effect of amino functionalization and C_60_ integration optimizes charge transfer processes, leading to an increased production of ROS that drive the photo‐Fenton reaction. P‐nitrosodimetilanilina (RNO) bleaching assays, which are widely used as a probe method for detecting •OH due to the selective reaction of •OH with RNO, were therefore employed to confirm the oxidative species generated in the system. RNO bleaching experiments (Figure 5c,d) demonstrated the effective generation of •OH radicals under both photocatalytic and photo‐Fenton conditions. In fact, the bleaching efficiency reached nearly 50% with C_60_@ZIF‐12‐NH_2_ after 120 min, while ZIF‐12 showed only marginal RNO degradation (<15%), and both ZIF‐12‐NH_2_ and C_60_@ZIF‐12 exhibited around 30%. These results indicate that •OH radicals are the main oxidative species responsible for contaminant degradation in the system, with their generation being markedly enhanced in the bifunctional composite. The higher RNO bleaching observed under photo‐Fenton compared to photocatalysis demonstrates the synergistic role of H_2_O_2_ activation in sustaining •OH production. Overall, the correlation between MB degradation and RNO bleaching strongly supports that the superior performance of C_60_@ZIF‐12‐NH_2_ is primarily governed by its enhanced ability to generate •OH, validating the proposed mechanism of action.

Building on these findings, the subsequent experiments assessed the disinfection efficiency of C_60_@ZIF‐12‐NH_2_ against bacteriophage P22 and against bacteria and coliforms present in natural river water samples. In these experiments, the H_2_O_2_ concentration was lowered from the 100 mg/L used during optimization to 10 mg/L, aiming to simulate more environmentally relevant conditions, reduce the need for external oxidants, and prevent possible secondary contamination. This modification allows for a more realistic evaluation of the catalyst's practical applicability in water treatment contexts.

### 2.3 Inactivation of P22 Phage

**FIGURE 6:**
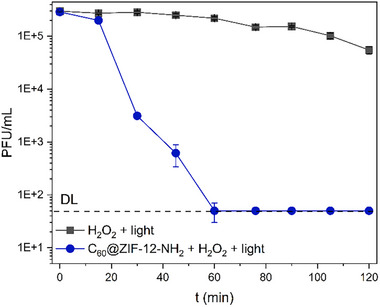
Inactivation of P22 bacteriophage using C_60_@ZIF‐12‐NH_2_ as catalyst by heterogeneous photo‐Fenton with 100 mg/L of H_2_O_2_, 0.05 g/L of catalyst, and 1032 W/cm^2^ of light irradiation.

Viral contaminants in water represent a major public health concern due to their persistence and resistance to conventional disinfection methods. Therefore, the hybrid catalyst C_60_@ZIF‐12‐NH_2_ was further evaluated for the inactivation of bacteriophage P22, a commonly used surrogate for *Salmonella*., spp., under heterogeneous photo‐Fenton conditions in simulated water. Figure 6 displays the inactivation kinetics of bacteriophage P22 using the hybrid catalyst C_60_@ZIF‐12‐NH_2_, H_2_O_2_ and visible light irradiation. The control experiment, which only included H_2_O_2_ and light, showed minimal antiviral activity over the 120 min‐period, with a reduction of less than 1 log_10_ in PFU/mL. This confirms that the combination of light and H_2_O_2_ alone is insufficient to generate the necessary ROS to induce effective viral inactivation. In contrast, the heterogeneous photo‐Fenton process using C_60_@ZIF‐12‐NH_2_ resulted in a rapid decrease of the viral concentration, achieving over 4 log_10_ reduction within the first 30 min. The waterborne virus concentration decreased to the detection limit (10^2^ PFU/mL) after 60 min and remained at this level for the rest of the experiment. The enhanced activity of C_60_@ZIF‐12‐NH_2_ can be attributed to the efficient electron transfer due to the encapsulation of C_60_ within the ZIF, mitigating charge recombination, a limitation noted for conventional semiconductors like TiO_2_ [112]. In addition, the amino group functionalization may improve viral adsorption onto the photocatalyst surface, a critical step for ROS damage, as demonstrated previously [111, 113], where these species oxidize the chemicals presented in the virus, such as the shell and capsid adsorbed on the photocatalyst surface [112]. Notably, the rapid inactivation kinetics is noteworthy when compared to the results of other reported studies. For instance, Rh‐SrTiO_3_ required 4 h for 5‐log Qb phage reduction under visible light [114], while Pd‐TiO_2_ achieved 2.8‐log airborne MS2 inactivation in 65 min under UV irradiation [115]. Although numerous studies have demonstrated the efficacy of MOFs for bacterial inactivation [116], there remains a lack of research exploring their application for viral disinfection, and no reported studies addressing the inactivation of P22 bacteriophage using MOFs have been found. This gap is particularly critical given that viruses, due to their small size, stability in water, and resistance to conventional treatments, pose significant challenges for water disinfection and public health protection.

### 2.4 River Water Disinfection

Given the promising results obtained in decontamination and disinfection in simulated water matrices, it is essential to evaluate the performance of the catalyst in natural water. Unlike controlled laboratory systems, natural waters contain a complex mixture of organic matter, inorganic ions, and diverse microbial populations that can interfere with ROS generation and reduce the overall efficiency of photocatalytic processes [117]. Therefore, testing under natural water conditions provides a more realistic assessment of the catalyst's disinfection capability and its potential applicability for practical water treatment. Prior to the disinfection experiments, the river water was physicochemically characterized to account for parameters that could influence the photocatalytic process, such as turbidity, conductivity, organic matter content, and chemical oxygen demand. The detailed water quality parameters are provided in Table S7.

The performance of the C_60_@ZIF‐12‐NH_2_ composite was further validated in water from a local river, where both *E. coli* and total coliforms were present at initial concentrations of approximately 10^2^–10^3^ CFU/100 mL (Table 1). After 2 h of heterogeneous photo‐Fenton treatment, bacterial colonies decreased up to the detection limit (1 CFU/100 mL), demonstrating complete inactivation of the microbial load. The efficient disinfection observed indicates that the ROS generated by the C_60_@ZIF‐12‐NH_2_ catalyst, mainly •OH radicals as previously evidenced, are sufficiently reactive and abundant to overcome the competing reactions within the complex natural‐water matrix. The synergy between C_60_ and the amino‐functionalized ZIF framework is likely responsible for the robust bactericidal activity. The use of real river water in this study provides an integrated assessment of the catalyst performance in the presence of natural organic matter and dissolved ionic species, which can potentially induce competitive adsorption, radical scavenging, or partial active‐site blockage. The sustained photocatalytic and disinfection activity observed under these conditions indicates that the C_60_@ZIF‐12‐NH_2_ composite is resilient to the combined effects of these parameters. A systematic investigation of individual water quality factors, such as pH, natural organic matter concentration, and ionic strength, would offer additional insight into process optimization and is therefore identified as an important direction for future work.

**TABLE 1:** River water disinfection with C_60_@ZIF‐12‐NH_2_ as catalyst by heterogeneous photo‐Fenton. Catalyst concentration = 0.05 g/L (C_60_ content = 12 mg), H_2_O_2_ concentration = 10 mg/L and irradiance = 1032 W/cm^2^.

**Microorganism**	**Pre‐treatment (CFU/100 mL)**	**Post‐treatment (CFU/100 mL)**
*E. coli*	∼1.2 × 10^2^	<DL (1 CFU/100 mL)
Total coliforms	∼1.0 × 10^3^	<DL (1 CFU/100 mL)

### 2.5 Recyclability and Stability of the ZIFs

**FIGURE 7:**
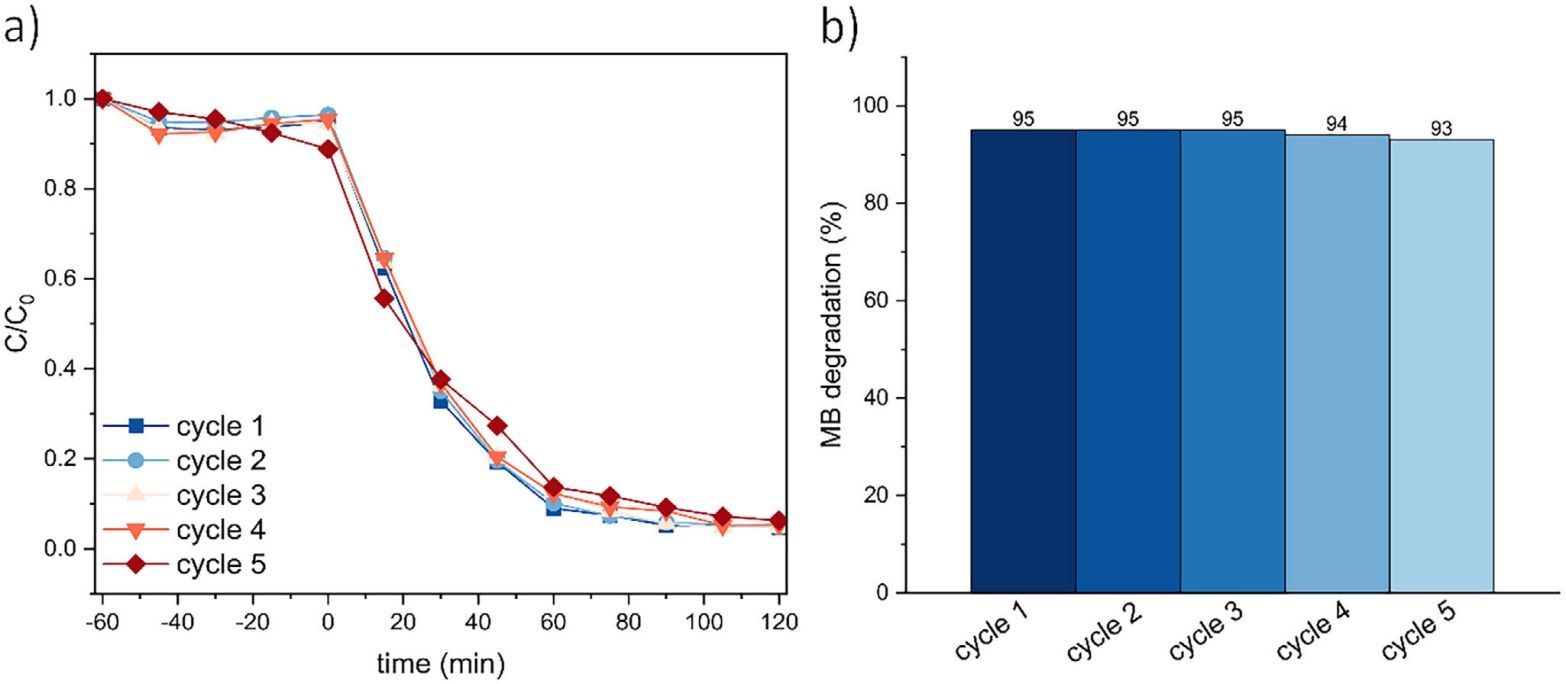
Recyclability kinetic of the degradation of MB under heterogeneous photo‐Fenton using C_60_@ZIF‐12‐NH_2_ as catalyst (a) and it respective MB degradation percentage over 5 cycles (b). MB concentration = 0.005 g/L, catalyst concentration = 0.05 g/L (C_60_ content12 mg for C_60_@ZIF‐12‐NH_2_), H_2_O_2_ concentration = 100 mg/L, irradiance = 1032 W/cm^2^.

The recyclability and reusability of the C_60_@ZIF‐12‐NH_2_ catalyst were assessed through five consecutive photocatalytic cycles for MB degradation under selected conditions (Figure 7). While minor variations in the rate of degradation can be observed, the overall kinetics remain largely unaltered, indicating the preservation of catalytic activity throughout the repeated use. Figure 7b further corroborates this observation by illustrating the degradation efficiencies at the end of each cycle. The catalyst maintains a high level of activity, remaining unaltered for five consecutive cycles. The sustained performance over multiple uses can be attributed to the robust framework of C_60_@ZIF‐12‐NH_2_.

**FIGURE 8:**
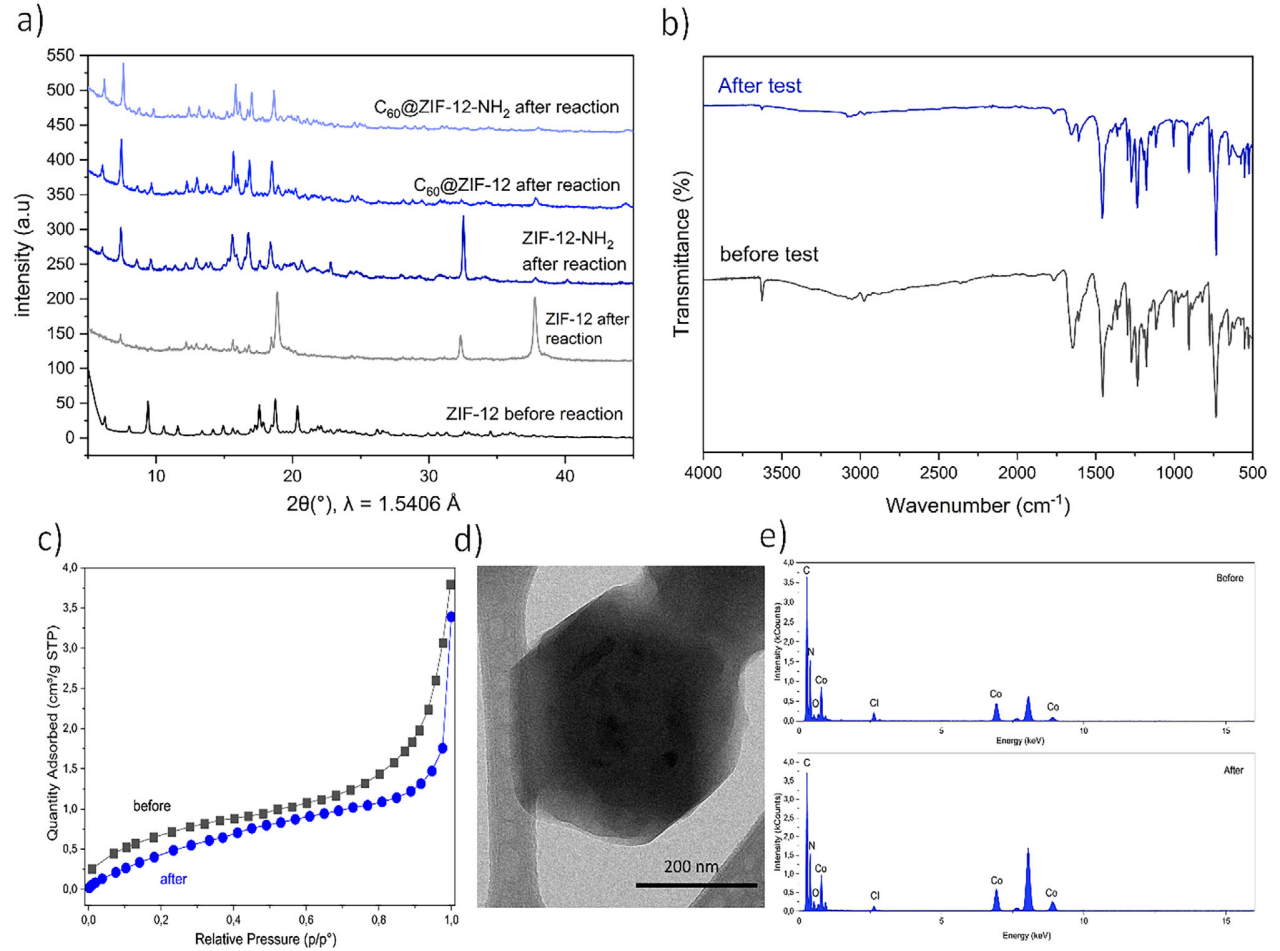
PXRD spectra of ZIF‐12, ZIF‐12‐NH_2_, C_60_@ZIF‐12, and C_60_@ZIF‐12‐NH_2_ (a) FT‐IR spectra of C_60_@ZIF‐12‐NH_2_ b), N_2_ adsorption isotherm of C_60_@ZIF‐12‐NH_2_ before and after cycling (c), TEM image of C_60_@ZIF‐12‐NH_2_ after cycling (d), and EDS data for the composition of C_60_@ZIF‐12‐NH_2_ particles before and after cycling under heterogenous photo‐Fenton process.

The PXRD analysis after the photocatalytic cycles revealed that some materials had significant structural differences compared to before use (Figure 8a). While ZIF‐12‐NH_2_, C_60_@ZIF‐12, and C_60_@ZIF‐12‐NH_2_ largely retain their crystallinity after MB degradation, ZIF‐12 exhibited notable degradation, showing the emergence of diffraction peaks associated with cobalt oxide species. This indicates that the ZIF‐12 framework is structurally less stable under photocatalytic conditions, likely due to its inherent susceptibility to hydrolysis and structural collapse in aqueous environments [118]. As reported in previous studies, monometallic MOFs such as ZIF‐12 suffer from poor water stability due to weak coordination bonds between metal ions and organic linkers, making them prone to framework decomposition [118]. In contrast, the superior structural preservation of C_60_@ZIF‐12 and C_60_@ZIF‐12‐NH_2_ can be attributed to the mechanochemical encapsulation of C_60_, which likely reinforces the framework and mitigates metal leaching. Additionally, the presence of the amino functional group in ZIF‐12‐NH_2_ and C_60_@ZIF‐12‐NH_2_ may enhance stability by modifying the electronic environment of the framework, improving resistance against hydrolysis, and maintaining active site integrity. FT‐IR spectra further confirm these results (Figure 8b), where the characteristic vibrational bands associated with the ZIF framework and organic linkers remain largely unchanged, indicating preservation of the chemical structure during the degradation process.

The structural robustness of C_60_@ZIF‐12‐NH_2_ was further supported by quantitative analysis of its textural and compositional stability post‐catalysis. Physisorption measurements revealed a high retention of porosity, with a very similar BET surface area of 2.35 m^2^ g^−^^1^ after cycling corroborating the preservation of the crystalline framework, in concordance with PXRD. N_2_ adsorption isotherm revealed 3.75 cm^3^/g (STP) and 3.40 cm^3^/g (STP) before and after cycling, respectively, confirming the retention of fullerene in the framework (Figure 8c). To rule out homogeneous catalysis via leached metal ions, the reaction solution was analyzed by ICP measurements. The concentration of cobalt was found to be below the detection limit (29 µg L^−^^1^), providing definitive evidence that the catalytic process is truly heterogeneous and that the ZIF matrix effectively retains the Co^2^^+^ centers.

The morphology and elemental distribution of the C_60_@ZIF‐12‐NH_2_ before and after photocatalytic cycling were compared by TEM coupled with EDX. As shown in Figure 8c, the material retains its structural integrity without signs of severe aggregation or morphological degradation, well‐defined particles with a characteristic morphology after cycling. In addition, similar compositions before and after cycling were observed (Figure 8d), containing approximately 73% of C, 20% of N2, and 6% of Co before and after the reaction (Table S8). These results provide direct visual evidence of the material's robustness. Complementary EDX elemental mapping confirmed a homogeneous distribution of cobalt throughout the particles in the used catalysts (Figure S9). The absence of cobalt‐rich aggregates or regions depleted of Co further supports the stability of the Co^2^^+^ centers within the ZIF framework and corroborates the negligible leaching detected by ICP analysis.

### 2.6 Comparative Analysis and Implications for Water Disinfection

The performance and novelty of the C_60_@ZIF‐12‐NH_2_ composite are best appreciated through a direct comparison with state‐of‐the‐art photocatalytic and photo‐Fenton systems for water disinfection, particularly in complex aqueous matrices. In recent years, various TiO_2_‐based photocatalysts have been demonstrated to inactivate *E. coli* under visible light irradiation. For instance, TiO_2_ anchored on natural pyrite effectively inactivated *E. coli* at neutral pH under visible light, likely via the generation of hydroxyl radical species [119]. Additionally, solar‐driven TiO_2_/Fe_2_O_3_ nanocomposites reached >97% *E. coli* inactivation in drinking water after 60 min [120]. Photocatalytic solar reactors with immobilized TiO_2_ have also shown significant reductions in natural water matrices, including *E. coli* and coliform bacteria, reaching values up to around 80% inactivation of coliform bacteria [121]. Similarly, g‐C_3_N_4_/TiO_2_ photocatalyst achieved the inactivation of 9.26 × 10^7^ cfu/(g·min) of *E. coli* under visible light after 180 min [122]. Sontakke, S. et al., evaluated the effect of different TiO_2_ based photocatalysts for *E. coli* inactivation under visible light, being Ag/TiO_2_> CS‐TiO_2_> Ag/TiO_2_ (Sub) > DP‐25, with a maximum efficiency of 6‐log reduction after 6 h [123]. Krumdieck et al. developed a nanostructured TiO_2_‐ based coating able to inactivate 3‐log of *E. coli* under visible light after 4 h [124].

While extensive research has been dedicated to the photocatalytic inactivation of bacteria such as *E. coli* in natural water matrices, the application of these systems for viral disinfection, particularly for the highly stable model bacteriophage P22, remains a significant and largely unexplored challenge. Notably, no reports were found describing the inactivation of bacteriophage P22 using either TiO_2_‐based systems or MOF‐based photocatalysts, highlighting a critical gap in the current literature on advanced oxidation processes for water safety. The performance of the present C_60_@ZIF‐12‐NH_2_ composite is noteworthy when benchmarked against reported photocatalytic systems. For viral inactivation, many visible‐light‐active TiO_2_ variants require extended treatment times; for instance, Rh‐SrTiO_3_ needed 120 min for a 5‐log reduction of Qβ phage [114], and Cu‐TiO_2_ achieved a 3.2‐log inactivation of bacteriophage f2 in 2 h [125]. Our system demonstrates competitive, rapid kinetics under visible light. Furthermore, photo‐Fenton process, highly effective for bacterial inactivation, also faces challenges in complex matrices. For example, Pino‐Sandoval et al., reported that, while a 6‐log reduction of *E. coli* and *S. typhimurium* was achieved in 120 min using iron‐oxide catalysts in distilled water, the inactivation rates in secondary‐treated wastewater effluent were 1.2‐5.9 times slower, requiring 150 min for complete elimination [126]. This underscores the significant matrix effect that real water imposes on even robust processes like photo‐Fenton. More broadly, while numerous studies have proven the efficacy of MOFs for bacterial inactivation, research exploring their specific application in river water and viral disinfection is scarce, and no prior studies addressing P22 bacteriophage inactivation with MOFs have been reported.

This work also builds upon and advances our group's previous developments in photocatalytic disinfection. We have successfully modified natural clinoptilolite with iron (NZ–Fe) and copper (NZ–Cu) for bacterial inactivation and developed a bimetallic Fe‐Cu clinoptilolite catalyst capable of inactivating natural river water bacteria to 1 CFU/100 mL within 60 min [127, 128]. Furthermore, UPO‐3, a ZIF‐9‐based photocatalyst, achieved a >5‐log reduction of *E. coli* and coliforms in river water [45]. C_60_@ZIF‐12‐NH_2_ composite represents a significant evolution by unifying several key advancements into a single material: (1) the efficient inactivation of both bacteria and, for the first time, the P22 bacteriophage under visible light; (2) the maintenance of high activity in complex, real river water matrices; and (3) the integration of a photosensitizer (C_60_) within an amino‐functionalized MOF to enhance visible‐light harvesting and charge separation. Therefore, the primary implication of this work is the introduction of a versatile photocatalytic platform with validated, dual‐mode functionality. It bridges a critical gap between a proven technology for real‐water bacterial disinfection and a pioneering capability for antiviral action. This combination addresses two of the most pressing concerns in water safety within a single, stable material. The composite's performance positions it as both a promising candidate for point‐of‐use systems targeting bacterial pathogens and a foundational material that opens new research avenues for the development of photocatalytic technologies capable of addressing the full spectrum of waterborne microbial threats, including resistant viruses.

## 3 Conclusions

This work demonstrates the successful mechanochemical synthesis of an amino‐functionalized ZIF‐12 framework with encapsulated fullerene, yielding a bifunctional composite active under visible‐light irradiation. The characterization of these materials confirmed the retention of C_60_ in the ZIF‐12 framework and its amino functionalization with variations in structural, morphological, and optical properties that support the effective incorporation of guest molecules. The synergistic interaction between amino groups and C_60_ enhances charge separation and •OH radicals’ generation, resulting in efficient photocatalytic degradation of organic contaminants and effective inactivation of bacteria and viruses, including bacteriophage P22.

Importantly, the material maintains its performance in river water, highlighting its robustness under environmentally relevant conditions. The solvent‐minimized mechanochemical approach, combined with visible‐light activity and broad‐spectrum disinfection capability, positions the C_60_@ZIF‐12‐NH_2_ composite as a promising candidate for advanced and sustainable water treatment applications, particularly as a quaternary treatment or disinfection technology.

These systems demonstrate recyclability and reusability, making them a potential platform for next‐generation photocatalysts with tunable properties adaptable to different contaminants and environmental conditions in water decontamination and disinfection. Future work for harnessing their potential for sustainable water purification should focus on validating these systems under real‐scale treatment conditions and testing their performance against individual water quality factors as well as assessing their effectiveness toward more recalcitrant pollutants and emerging contaminants.

## 4 Experimental Section

### 4.1 Materials

Benzimidazole (bml, ≥ 99%), Cobalt (II) hydroxide (Co(OH)_2_, ≥ 95%), and MB were obtained from Sigma Aldrich. Fullerene and 2‐aminobezimidazole were purchased from Carl Roth. Ammonium chloride (NH_4_Cl, ≥ 99%) was purchased from Fisher Scientific. hydrogen peroxide, N,N‐diethylformamide, toluene, and ammonium hydroxide 25% (NH_4_OH) were acquired from ChemSolute.

### 4.2 Characterization

The structural and morphological characterization of ZIF‐12, C_60_@ZIF‐12, ZIF‐12‐NH_2_, and C_60_@ZIF‐12‐NH_2_ was conducted using multiple analytical techniques. X‐ray diffraction (XRD) analysis was performed with a Bruker D8 Advance diffractometer, while Fourier transform infrared (FTIR) spectroscopy measurements were obtained using a Thermo Nicolet NEXUS 470 FTIR spectrophotometer. To assess the thermal stability of the samples, thermogravimetric analysis (TGA) was carried out under a nitrogen atmosphere using a Thermal Analysis System TGA/DSC 3+ instrument. The morphological characteristics of the materials were examined through Scanning Electron Microscopy (SEM) using an XL30 ESEM, while Transmission Electron Microscopy (TEM) images were captured with a Talos F200S microscope (Thermo Fisher Scientific). In TEM mode, a Ceta 16 M camera was employed, whereas in Scanning Transmission Electron Microscopy (STEM) mode, images were acquired using a High‐Angle Annular Dark Field (HAADF) and Bright Field (BF) detector. Additionally, Energy Dispersive X‐ray Spectroscopy (EDS) was performed with a Super‐X G2 detector equipped with two silicon drift detectors (SDD) to determine the elemental composition of the samples. Brunauer‐Emmett‐Teller (BET) surface area analyses were conducted using an ASAP instrument from Micromeritics Instrument Corporation, with nitrogen adsorption isotherms measured at 77 K. The specific surface areas (ABET) were calculated in a relative pressure range of 0.04 ≤ p/p0< 0.22 using the multipoint BET method with at least five supporting data points. DRS measurements were obtained using a UV–vis–NIR PerkinElmer Lambda 950 spectrophotometer. Band Gap was obtained from Tauc plot, for further details, see S.I. ICP‐MS measurements were performed using an ICP‐MS/MS Agilent 8800.

### 4.3 Synthesis of ZIF‐12

1 mm of bml (0,118 g), 0.5 mm of cobalt (II) hydroxide (0,046 g), and 0,13 mm of NH_4_Cl (7 mg) were mixed with 100 µL of DEF and 100 µL of Toluene in a 10 mL stainless steel jar with 2 × 7 mm stainless steel ball for horizontal ball milling. The mixture was milled for 90 min at a rate of 30 Hz using a Retsch MM400 shaker‐type mixer mill. The powder was collected scratching the wall of the jar. Finally, the MOF was washed with MeOH, filtered, and dried at room temperature. The final molar ratio used for the synthesis was 1:0.5:0.13:1.2:0.3 for the reagents bml:Co(OH)_2_:NH_4_Cl:DEF:Tol

### 4.4 Synthesis of C_60_@ZIF‐12

1 mm of bml (0,118 g), 0.5 mm of cobalt (II) hydroxide (0,046 g), 6 (∼2 wt%,0.0083 mmol), 9 (∼3 wt%, 0.0124 mmol), 12 (∼4 wt%, 0.0166 mmol) or 15 mg (∼5 wt%, 0.0208 mmol) of C_60_ fullerene and 0,37 mm of NH_4_Cl (20 mg) were mixed with 100 µL of DEF and 100 µL of Toluene in a 10 mL stainless steel jar with 2 × 7 mm stainless steel ball for horizontal ball milling. The mixture was milled for 90 min at a rate of 30 Hz using a Retsch MM400 shaker‐type mixer mill for horizontal milling. The powder was collected scratching the wall of the jar. Finally, the MOF was washed with Toluene, filtered, and dried at room temperature. The final molar ratio used for the synthesis was 1:0.5:0.37:1.2:0.3 for the reagents bml:Co(OH)_2_:NH_4_Cl:DEF:Tol.

### 4.5 Synthesis of ZIF‐12‐NH_2_

0.9 mm of bml (0,106 g), 0.5 mm of cobalt (II) hydroxide (0,046 g), 0.1 mmol of 2‐NH_2_‐bml, and 0,37 mm of NH_4_Cl (20 mg) were mixed with 100 µL of DEF and 100 µL of Toluene in a 10 mL stainless steel jar with 2 × 7 mm stainless steel ball for horizontal ball milling. The mixture was milled for 90 min at a rate of 30 Hz using a Retsch MM400 shaker‐type mixer mill. The powder was collected by scratching the wall of the jar. Finally, the MOF was washed with MeOH, filtered, and dried at room temperature. The final molar ratio used for the synthesis was 0.9:0.1:0.5:0.37:0.5:1.2:0.3 for the reagents bml:2‐bml‐NH_2_:Co(OH)_2_:NH_4_Cl:DEF:Tol.

### 4.6 Synthesis of C_60_@ZIF‐12‐NH_2_

0.9 mm of bml (0,106 g), 0.5 mm of cobalt (II) hydroxide (0,046 g), 6 (∼2 wt%,0.0083 mmol), 9 (∼3 wt%, 0.0124 mmol), 12 (∼4 wt%, 0.0166 mmol) or 15 mg (∼5 wt%, 0.0208 mmol) of C_60_, 0.1 mmol of 2‐NH_2_‐bml and 0,37 mm of NH_4_Cl (20 mg) were mixed with 100 µL of DEF and 100 µL of Toluene in a 10 mL stainless steel jar with 2 × 7 mm stainless steel ball for horizontal milling. The mixture was milled for 90 min at a rate of 30 Hz using a Retsch MM400 shaker‐type mixer mill. The powder was collected scratching the wall of the jar Finally, the MOF was washed with Toluene, filtered, and dried at room temperature. The final molar ratio used for the synthesis was 0.9:0.1:0.5:0.37:1.2:0.3 for the reagents bml:2‐bml‐NH_2_:Co(OH)_2_:NH_4_Cl:DEF:Tol

### 4.7 Evaluation of the Photocatalytic Activity

To assess the photocatalytic performance of the catalyst, a MB solution (50 mL, 5 mg/L) and 0.5 g/L of the catalyst were introduced into the photoreactor and kept in darkness for 60 min under continuous stirring at 600 rpm, maintaining a temperature of 25°C and a neutral pH. This step allowed the adsorption of MB onto the MOF to be monitored until equilibrium was reached. Subsequently, the light source (1032 W/cm^2^) was then activated, and irradiation was maintained for 120 min. Samples were collected every 15 min, centrifuged, and their absorbance was measured using a UV–vis spectrophotometer at 664 nm for MB. To prevent catalyst loss or changes in solution volume and ensure accuracy, all samples were returned to the reactor. Each experiment was conducted in triplicate to verify reproducibility. For the degradation of MB by photo‐Fenton processes, the experiments were carried out following the same procedure but with the addition of H_2_O_2_ immediately after turning on the lights, to achieve an initial concentration of 10 mm (35 µL), as the standard method that we described before [129]. The light source was then activated, and irradiation was maintained for 120 min. ZIFs were recovered by centrifuging the final solution and drying them in an oven at 60°C for 2 h. XRD spectroscopy analysis was performed to assess the structural stability of the ZIFs. Recyclability of the optimal C_60_@ZIF‐12‐NH_2_ was also evaluated during 5 consecutive cycles under the same conditions. Detailed methodology for adsorption kinetics and degradation kinetics can be found in the S.I.

### 4.8 Inactivation of P22 Bacteriophage and River Water Disinfection

Disinfection tests were performed using an initial P22 sppHT105/1 int‐201 bacteriophage concentration of approximately 10^5^ PFU/mL. The tests were performed using 100 mg/L of H_2_O_2_, 0.05 g/L of catalyst, and an irradiance of 1032 W/m^2^. The 50 mL reactor was stirring at 600 rpm for 120 min, and each sample was taken every 15 min with the addition of 0.1 g/L of Bovine catalase to eliminate any residual H_2_O_2_ and plated immediately after sampling. In addition, a sample was taken before each disinfection test to confirm the initial concentration of P22 bacteriophage in the reactor. During the experiments, samples were quantified in triplicate using the standard plate count method after performing 10‐fold serial dilutions in LB broth containing an *E. coli* culture. The plates were incubated at 37°C for 24 h. Each sample was processed in triplicate with a detection limit of 50 PFU/mL for this method. For river water disinfection test, the water was taken from Guadaíra River, Seville, Spain and settled for decantation for 24 h. The treatment was performed using 100 mg/L of H_2_O_2_, 0.05 g/L of catalyst and an irradiance of 1032 W/m^2^ for 120 min. *E. coli* and coliforms were quantified prior to treatment by directly inoculating 1 mL of the sample in a Chromatic Coliform Agar ISO plate (chromogenic medium) and incubated aerobically at 36 ± 2°C for 24 h. Following treatment, 100 mL of sample was filtered using a 0.45 µm pore‐size membrane filter, which was the placed directly onto a plate containing the same medium and incubated under identical conditions. The detection limit was 1 CFU per 100 mL.

## Funding

This project was funded by VALZEO project from the European Commission (HORIZON MSCA‐2021‐SE‐01 under grant agreement no. 101086354) and the Programa de Excelencia de la Junta de Andalucía (ProyExcel_00358) granted to I.C and A.F.

## Conflicts of Interest

The authors declare no conflicts of interest.

## Supporting information




**Supporting File**: smll72928‐sup‐0001‐SuppMat.docx.

## Data Availability

The data that support the findings of this study are available from the corresponding author upon reasonable request.

## References

[1] I. Corsi , I. Venditti , F. Trotta , and C. Punta , “Environmental Safety of Nanotechnologies: The Eco‐design of Manufactured Nanomaterials for Environmental Remediation,” Science of The Total Environment 864 (2023): 161181, 10.1016/j.scitotenv.2022.161181. 36581299

[2] M. Patel , R. Kumar , K. Kishor , T. Mlsna , C. U. Pittman , and D. Mohan , “Pharmaceuticals of Emerging Concern in Aquatic Systems: Chemistry, Occurrence, Effects, and Removal Methods,” Chemical Reviews 119 (2019): 3510–3673, 10.1021/acs.chemrev.8b00299. 30830758

[3] Y. Yang , Y. S. Ok , K.‐H. Kim , E. E. Kwon , and Y. F. Tsang , “Occurrences and Removal of Pharmaceuticals and Personal Care Products (PPCPs) in Drinking Water and Water/Sewage Treatment Plants: A Review,” Science of The Total Environment 596‐597 (2017): 303–320, 10.1016/j.scitotenv.2017.04.102. 28437649

[4] R. M. De Souza , D. Seibert , H. B. Quesada , F. De Jesus Bassetti , M. R. Fagundes‐Klen , and R. Bergamasco , “Occurrence, Impacts and General Aspects of Pesticides in Surface Water: a Review,” Process Safety and Environmental Protection 135 (2020): 22–37, 10.1016/j.psep.2019.12.035.

[5] S. G. Goh , N. Saeidi , X. Gu , et al., “Occurrence of Microbial Indicators, Pathogenic Bacteria and Viruses in Tropical Surface Waters Subject to Contrasting Land Use,” Water Research 150 (2019): 200–215, 10.1016/j.watres.2018.11.058. 30528917 PMC7112093

[6] N. H. Tran , M. Reinhard , and K. Y.‐H. Gin , “Occurrence and Fate of Emerging Contaminants in Municipal Wastewater Treatment Plants from Different Geographical Regions‐a Review,” Water Research 133 (2018): 182–207, 10.1016/j.watres.2017.12.029. 29407700

[7] S. Gabarrón , W. Gernjak , F. Valero , A. Barceló , M. Petrovic , and I. Rodríguez‐Roda , “Evaluation of Emerging Contaminants in a Drinking Water Treatment Plant Using Electrodialysis Reversal Technology,” Journal of Hazardous Materials 309 (2016): 192–201, 10.1016/j.jhazmat.2016.02.015. 26894293

[8] T. Nikbeen and A. K. Nayab , “Transformation of Traditional Wastewater Treatment Methods into Advanced Oxidation Processes and the Role of Ozonation,” Journal of Ecological Engineering 24 (2023): 173–189, 10.12911/22998993/162777.

[9] S. Garcia‐Segura , et al., “Opportunities for nanotechnology to enhance electrochemical treatment of pollutants in potable water and industrial wastewater–a perspective,” Environmental Science: Nano, 7 (2020): 2178–2194, 10.1039/D0EN00194E.

[10] R. Kuhn , I. M. Bryant , R. Jensch , and J. Böllmann , “Applications of Environmental Nanotechnologies in Remediation, Wastewater Treatment, Drinking Water Treatment, and Agriculture,” Applied Nano 3 (2022): 54–90, 10.3390/applnano3010005.

[11] S. Zhang , T. Hedtke , X. Zhou , M. Elimelech , and J.‐H. Kim , “Environmental Applications of Engineered Materials with Nanoconfinement,” ACS ES&T Engineering 1 (2021): 706–724, 10.1021/acsestengg.1c00007.

[12] V. Sodha , S. Shahabuddin , R. Gaur , I. Ahmad , R. Bandyopadhyay , and N. Sridewi , “Comprehensive Review on Zeolite‐Based Nanocomposites for Treatment of Effluents from Wastewater,” Nanomaterials 12 (2022): 3199, 10.3390/nano12183199. 36144986 PMC9504493

[13] N. A. Khan , Z. Hasan , and S. H. Jhung , “Adsorptive Removal of Hazardous Materials Using Metal‐organic Frameworks (MOFs): A Review,” Journal of Hazardous Materials 244‐245 (2013): 444–456, 10.1016/j.jhazmat.2012.11.011. 23195596

[14] S. Rojas and P. Horcajada , “Metal–Organic Frameworks for the Removal of Emerging Organic Contaminants in Water,” Chemical Reviews 120 (2020): 8378–8415, 10.1021/acs.chemrev.9b00797. 32023043

[15] N. Viet , T. Ha , and N. Tri , Metal Organic Frameworks for Wastewater Contaminant Removal, 1st ed., edited by A. L. Srivastav , L. Rani , J. Kaushal , and T. D. Pham , (Wiley, 2023), 1–25, 10.1002/9783527841523.ch5.

[16] M. M. H. Mondol and S. H. Jhung , “Adsorptive Removal of Pesticides from Water with Metal–organic Framework‐based Materials,” Chemical Engineering Journal 421 (2021): 129688, 10.1016/j.cej.2021.129688.

[17] S. Abbas , K. Ahmad , K. Naseem , et al., “Cutting‐edge Metal‐organic Frameworks: Revolutionizing the Adsorptive Removal of Pharmaceutical Contaminants from Water,” Reviews in Inorganic Chemistry 45: 885–905, 10.1515/revic-2024-0119.

[18] B. Yao and Y. Zhou , “Removal of Neonicotinoid Insecticides from Water in Various Treatment Processes: A Review,” Critical Reviews in Environmental Science and Technology 54 (2024): 1307–1339, 10.1080/10643389.2024.2309845.

[19] R. Li , N. N. Adarsh , H. Lu , and M. Wriedt , “Metal‐organic Frameworks as Platforms for the Removal of Per‐and Polyfluoroalkyl Substances from Contaminated Waters,” Matter 5 (2022): 3161–3193, 10.1016/j.matt.2022.07.028.

[20] K. Solanki , S. Sharma , S. Yadav , et al., “Hierarchical 3D Flower‐like Metal Oxides Micro/Nanostructures: Fabrication, Surface Modification, Their Crucial Role in Environmental Decontamination, Mechanistic Insights, and Future Perspectives,” Small 19 (2023): 2300394, 10.1002/smll.202300394. 36950767

[21] Q. Gao , J. Xu , and X.‐H. Bu , “Recent Advances about Metal–organic Frameworks in the Removal of Pollutants from Wastewater,” Coordination Chemistry Reviews 378 (2019): 17–31, 10.1016/j.ccr.2018.03.015.

[22] C. Du , Z. Zhang , G. Yu , et al., “A Review of Metal Organic Framework (MOFs)‐based Materials for Antibiotics Removal via Adsorption and Photocatalysis,” Chemosphere 272 (2021): 129501, 10.1016/j.chemosphere.2020.129501. 33486457

[23] D. B. Miklos , C. Remy , M. Jekel , K. G. Linden , J. E. Drewes , and U. Hübner , “Evaluation of Advanced Oxidation Processes for Water and Wastewater Treatment—A Critical Review,” Water Research 139 (2018): 118–131, 10.1016/j.watres.2018.03.042. 29631187

[24] H. Wang , Y. Wu , M. Feng , et al., “Visible‐light‐driven Removal of Tetracycline Antibiotics and Reclamation of Hydrogen Energy from Natural Water Matrices and Wastewater by Polymeric Carbon Nitride Foam,” Water Research 144 (2018): 215–225, 10.1016/j.watres.2018.07.025. 30031366

[25] Y. Zhang , J. Zhou , X. Chen , L. Wang , and W. Cai , “Coupling of Heterogeneous Advanced Oxidation Processes and Photocatalysis in Efficient Degradation of Tetracycline Hydrochloride by Fe‐based MOFs: Synergistic Effect and Degradation Pathway,” Chemical Engineering Journal 369 (2019): 745–757, 10.1016/j.cej.2019.03.108.

[26] X. Hua , H. Chen , C. Rong , et al., “Visible‐light‐driven Photocatalytic Degradation of Tetracycline Hydrochloride by Z‐scheme Ag_3_PO_4_/1T@2H‐MoS_2_ Heterojunction: Degradation Mechanism, Toxicity Assessment, and Potential Applications,” Journal of Hazardous Materials 448 (2023): 130951, 10.1016/j.jhazmat.2023.130951. 36860039

[27] S. Malato , J. Blanco , M. I. Maldonado , I. Oller , W. Gernjak , and L. Pérez‐Estrada , “Coupling Solar Photo‐Fenton and Biotreatment at Industrial Scale: Main Results of a Demonstration Plant,” Journal of Hazardous Materials 146 (2007): 440–446, 10.1016/j.jhazmat.2007.04.084. 17532127

[28] S. Malato , M. I. Maldonado , P. Fernández‐Ibáñez , I. Oller , I. Polo , and R. Sánchez‐Moreno , “Decontamination and Disinfection of Water by Solar Photocatalysis: The Pilot Plants of the Plataforma Solar de Almeria,” Materials Science in Semiconductor Processing 42 (2016): 15–23, 10.1016/j.mssp.2015.07.017.

[29] X. Li , J. Xie , C. Jiang , J. Yu , and P. Zhang , “Review on Design and Evaluation of Environmental Photocatalysts,” Frontiers of Environmental Science & Engineering 12 (2018): 14, 10.1007/s11783-018-1076-1.

[30] S. Lu , L. Liu , H. Demissie , G. An , and D. Wang , “Design and Application of Metal‐organic Frameworks and Derivatives as Heterogeneous Fenton‐Like Catalysts for Organic Wastewater Treatment: A Review,” Environment International 146 (2021): 106273, 10.1016/j.envint.2020.106273. 33264734

[31] Z. Wang , Y. Cheng , C. Wang , R. Guo , J. You , and H. Zhang , “Optimizing the Performance of Fe‐based Metal‐organic Frameworks in Photo‐Fenton Processes: Mechanisms, Strategies and Prospects,” Chemosphere 339 (2023): 139673, 10.1016/j.chemosphere.2023.139673. 37536536

[32] S. A. Ahmed , D. Bagchi , H. A. Katouah , M. N. Hasan , H. M. Altass , and S. K. Pal , “Enhanced Water Stability and Photoresponsivity in Metal‐Organic Framework (MOF): A Potential Tool to Combat Drug‐resistant Bacteria,” Scientific Reports 9 (2019): 19372, 10.1038/s41598-019-55542-8. 31852949 PMC6920456

[33] N. Talukder , Y. Wang , B. B. Nunna , X. Tong , and E. S. Lee , “An Investigation on the Structural Stability of ZIF‐8 in Water versus Water‐derived Oxidative Species in Aqueous Environment,” Microporous and Mesoporous Materials 366 (2024): 112934, 10.1016/j.micromeso.2023.112934.

[34] S. Kouser , A. Hezam , M. J. N. Khadri , and S. A. Khanum , “A Review on Zeolite Imidazole Frameworks: Synthesis, Properties, and Applications,” Journal of Porous Materials 29 (2022): 663–681, 10.1007/s10934-021-01184-z.

[35] H. Wang , X. Pei , M. J. Kalmutzki , J. Yang , and O. M. Yaghi , “Large Cages of Zeolitic Imidazolate Frameworks,” Accounts of Chemical Research 55 (2022): 707–721, 10.1021/acs.accounts.1c00740. 35170938

[36] Z. Zheng , Z. Rong , H. L. Nguyen , and O. M. Yaghi , “Structural Chemistry of Zeolitic Imidazolate Frameworks,” Inorganic Chemistry 62 (2023): 20861–20873, 10.1021/acs.inorgchem.3c02322. 38063312

[37] Y. Sun , N. Zhang , Y. Yue , J. Xiao , X. Huang , and A. Ishag , “Recent Advances in the Application of Zeolitic Imidazolate Frameworks (ZIFs) in Environmental Remediation: a Review,” Environmental Science: Nano 9 (2022): 4069–4092, 10.1039/D2EN00601D.

[38] Y. Zhang , Y. Sun , Y. Man , et al., “Highly Efficient Adsorption and Catalytic Degradation of Aflatoxin B1 by a Novel Porous Carbon Material Derived from Fe‐doped ZIF‐8,” Chemical Engineering Journal 440 (2022): 135723, 10.1016/j.cej.2022.135723.

[39] S. Zhao , Y. Zhang , Y. Wu , L. Zhang , H. Hu , and L. Jin , “ZIF‐derived Hierarchical Pore Carbons as High‐performance Catalyst for Methane Decomposition,” Journal of the Energy Institute 100 (2022): 197–205, 10.1016/j.joei.2021.11.015.

[40] Y. V. Kaneti , S. Dutta , M. S. A. Hossain , et al., “Strategies for Improving the Functionality of Zeolitic Imidazolate Frameworks: Tailoring Nanoarchitectures for Functional Applications,” Advanced Materials 29 (2017): 1700213, 10.1002/adma.201700213. 28833624

[41] W. Xie and F. Wan , “Guanidine Post‐functionalized Crystalline ZIF‐90 Frameworks as a Promising Recyclable Catalyst for the Production of Biodiesel via Soybean Oil Transesterification,” Energy Conversion and Management 198 (2019): 111922, 10.1016/j.enconman.2019.111922.

[42] L.‐X. You , B.‐B. Zhao , S.‐X. Yao , et al., “Engineering Functional Group Decorated ZIFs to High‐performance Pd@ZIF‐92 Nanocatalysts for C(sp^2^)‐C(sp^2^) Couplings in Aqueous Medium,” Journal of Catalysis 392 (2020): 80–87, 10.1016/j.jcat.2020.09.024.

[43] R. Grau‐Crespo , A. Aziz , A. W. Collins , et al., “Modelling a Linker Mix‐and‐Match Approach for Controlling the Optical Excitation Gaps and Band Alignment of Zeolitic Imidazolate Frameworks,” Angewandte Chemie International Edition 55 (2016): 16012–16016, 10.1002/anie.201609439. 27862763 PMC5216902

[44] L. Santos‐Juanes , N. Rodriguez‐Sanchez , S. R. G. Balestra , et al., “A Hypervalent Metal MOF Catalyst as an Avenue to Go beyond Heterogeneous Fenton‐Like Processes for Organic Contaminant Removal in Water,” Materials Advances 6 (2025): 3612–3621, 10.1039/D4MA01217H.

[45] N. Rodríguez‐Sánchez , J. E. Domínguez‐Santos , B. Bhattacharya , et al., “Mechanochemical Synthesis of Multivariate UPO‐3 (Cu‐ZIF‐9‐ica) MOF for Inactivation of Antibiotic‐resistant Bacteria and Irrigation‐quality Water Production via Heterogeneous Photo‐Fenton Catalysis,” Chemosphere 386 (2025): 144610, 10.1016/j.chemosphere.2025.144610. 40818911

[46] Z. Xu , Y. Wang , Y. Li , et al., “C_60_ and Derivatives Boost Electrocatalysis and Photocatalysis: Electron Buffers to Heterojunctions,” Advanced Energy Materials 13 (2023): 2302438, 10.1002/aenm.202302438.

[47] X. Chang , Y. Xu , and M. Von Delius , “Recent Advances in Supramolecular Fullerene Chemistry,” Chemical Society Reviews 53 (2024): 47–83, 10.1039/D2CS00937D. 37853792 PMC10759306

[48] V. Martinez , B. Karadeniz , N. Biliškov , et al., “Tunable Fulleretic Sodalite MOFs: Highly Efficient and Controllable Entrapment of C 60 Fullerene via Mechanochemistry,” Chemistry of Materials 32 (2020): 10628–10640, 10.1021/acs.chemmater.0c03796.

[49] M. He , J. Yao , Q. Liu , Z. Zhong , and H. Wang , “Toluene‐assisted Synthesis of RHO‐type Zeolitic Imidazolate Frameworks: Synthesis and Formation Mechanism of ZIF‐11 and ZIF‐12,” Dalton Transactions 42 (2013): 16608, 10.1039/c3dt52103f. 24071923

[50] A. Noguera‐Díaz , J. Villarroel‐Rocha , V. P. Ting , N. Bimbo , K. Sapag , and T. J. Mays , “Flexible ZIFs: Probing Guest‐Induced Flexibility with CO_2_, N_2_ and Ar Adsorption,” Journal of Chemical Technology & Biotechnology 94 (2019): 3787–3792, 10.1002/jctb.5947.

[51] K. Alhazzani , A. Z. Alanazi , H. Ibrahim , et al., “ZIF‐12‐derived Nitrogen Doped Porous Carbon Nanosheets‐functionalized with Silver Nanoparticles for Ratiometric Molecularly‐imprinted Electrochemical Sensing of D‐penicillamine,” Microchemical Journal 206 (2024): 111635, 10.1016/j.microc.2024.111635.

[52] Y. Miao , D. T. Lee , M. D. De Mello , et al., “Solvent‐free Bottom‐up Patterning of Zeolitic Imidazolate Frameworks,” Nature Communications 13 (2022): 420, 10.1038/s41467-022-28050-z. PMC8776825 35058452

[53] F. Afshariazar and A. Morsali , “The Unique Opportunities of Mechanosynthesis in Green and Scalable Fabrication of Metal–organic Frameworks,” Journal of Materials Chemistry A 10 (2022): 15332–15369, 10.1039/D2TA02699F.

[54] S. Głowniak , B. Szczęśniak , J. Choma , and M. Jaroniec , “Mechanochemical Synthesis of MOF‐303 and Its CO_2_ Adsorption at Ambient Conditions,” Molecules 29 (2024): 2698, 10.3390/molecules29112698. 38893571 PMC11173739

[55] T. Friščić , “New Opportunities for Materials Synthesis Using Mechanochemistry,” Journal of Materials Chemistry 20 (2010): 7599–7605, 10.1039/C0JM00872A.

[56] S. L. James , C. J. Adams , C. Bolm , et al., “Mechanochemistry: Opportunities for New and Cleaner Synthesis,” Chemical Society Reviews 41 (2012): 413–447, 10.1039/C1CS15171A. 21892512

[57] H. K. Chae , D. Y. Siberio‐Pérez , J. Kim , et al., “Materials Design and Discovery Group. A Route to High Surface Area, Porosity and Inclusion of Large Molecules in Crystals,” Nature 427 (2004): 523–527, 10.1038/nature02311. 14765190

[58] T. Friščić , I. Halasz , P. J. Beldon , et al., “Real‐time and in situ monitoring of mechanochemical milling reactions,” Nature Chem 5 (2013): 66–73, 10.1038/nchem.1505. 23247180

[59] D. W. Lewis , A. R. Ruiz‐Salvador , A. Gómez , et al., “Zeolitic Imidazole Frameworks: Structural and Energetics Trends Compared with Their Zeolite Analogues,” CrystEngComm 11 (2009): 2272, 10.1039/b912997a.

[60] J. T. Hughes , T. D. Bennett , A. K. Cheetham , and A. Navrotsky , “Thermochemistry of Zeolitic Imidazolate Frameworks of Varying Porosity,” Journal of the American Chemical Society 135 (2013): 598–601, 10.1021/ja311237m. 23270310

[61] P. J. Beldon , L. Fábián , R. S. Stein , A. Thirumurugan , A. K. Cheetham , and T. Friščić , “Rapid Room‐Temperature Synthesis of Zeolitic Imidazolate Frameworks by Using Mechanochemistry,” Angewandte Chemie International Edition 49 (2010): 9640–9643, 10.1002/anie.201005547. 21077080

[62] N. Rodríguez‐Sánchez , C. Prinz , R. Bienert , et al., “Mechanochemical ZIF‐9 Formation: in Situ Analysis and Photocatalytic Enhancement Evaluation,” RSC Mechanochemistry 2 (2025): 116–126, 10.1039/D4MR00114A.

[63] M. Abdolmaleki , A. Dehghani , N. Alipanah , G. Bahlakeh , P. Haghdadeh , and B. Ramezanzadeh , “ZIF‐12 Nano‐porous Frameworks for Designing Double Stimuli‐responsive Smart Anticorrosion Coating Inherited Superior Mechanical Properties; Experimental and Modeling Studies,” Applied Materials Today 35 (2023): 101965, 10.1016/j.apmt.2023.101965.

[64] W. Guo , J. Liu , P. G. Weidler , et al., “Loading of Ionic Compounds into Metal–organic Frameworks: A Joint Theoretical and Experimental Study for the Case of La^3+^ ,” Physical Chemistry Chemical Physics 16 (2014): 17918–17923, 10.1039/C4CP02285H. 25046605

[65] L. Sondermann , Q. Smith , T. Strothmann , A. Vollrath , T. H. Yen Beglau , and C. Janiak , “Mechanochemical Synthesis and Application of Mixed‐metal Copper–ruthenium HKUST‐1 Metal–organic Frameworks in the Electrocatalytic Oxygen Evolution Reaction,” RSC Mechanochemistry 1 (2024): 296–307, 10.1039/D4MR00021H.

[66] J. Abdi , F. Banisharif , and A. Khataee , “Amine‐functionalized Zr‐MOF/CNTs Nanocomposite as an Efficient and Reusable Photocatalyst for Removing Organic Contaminants,” Journal of Molecular Liquids 334 (2021): 116129, 10.1016/j.molliq.2021.116129.

[67] J. Dong , S.‐Q. Wang , X.‐Y. Zhang , Z.‐Q. Huang , X.‐D. Zhang , and W.‐Y. Sun , “Turn‐on Fluorescence Detection of Specific Inorganic Anions by Zr(IV)‐MOF with Amino‐functional Group,” Tungsten 5 (2023): 217–224, 10.1007/s42864-022-00198-7.

[68] T. Thenrajan , M. Madhu Malar , and J. Wilson , “Natural Polymer Encapsulated Zeolitic Imidazolate Framework‐12 Composite toward Electrochemical Sensing of Antitumor Agent,” ACS Applied Bio Materials 7 (2024): 3375–3387, 10.1021/acsabm.4c00314. 38693867

[69] A. D. Ali , R. F. Shafi , W. N. Khudhair , and S. H. Ammar , “Metals ferrite decorated in metal organic framework (Mn0. 5Zn0. 5Fe2O4/ZIF‐12) for adsorption of organic acid red dye,” AIP Conference Proceedings 3079, 060036 (2024), 10.1063/5.0207397.

[70] R. Ghamarpoor , A. Fallah , N. E. Fard , S. Salehfekr , S. Hossieini , and V. Moradi , “Multifunctional Nanocomposite of TiO_2_‐decorated on Graphitic Carbon Derived from ZIF‐12: Towards Enhanced Photocatalytic Degradation and Supercapacitor Performance,” Surfaces and Interfaces 76 (2025): 107846, 10.1016/j.surfin.2025.107846.

[71] S.‐N. Kim , J. Kim , H.‐Y. Kim , H.‐Y. Cho , and W.‐S. Ahn , “Adsorption/Catalytic Properties of MIL‐125 and NH2‐MIL‐125,” Catalysis Today 204 (2013): 85–93, 10.1016/j.cattod.2012.08.014.

[72] I. Ahmed and S. H. Jhung , “Effective Adsorptive Removal of Indole from Model Fuel Using a Metal‐organic Framework Functionalized with Amino Groups,” Journal of Hazardous Materials 283 (2015): 544–550, 10.1016/j.jhazmat.2014.10.002. 25464294

[73] R. Ding , W. Zheng , K. Yang , et al., “Amino‐functional ZIF‐8 Nanocrystals by Microemulsion Based Mixed Linker Strategy and the Enhanced CO_2_/N_2_ Separation,” Separation and Purification Technology 236 (2020): 116209, 10.1016/j.seppur.2019.116209.

[74] S. Tian , S. Xu , J. Liu , C. He , Y. Xiong , and P. Feng , “Highly Efficient Removal of both Cationic and Anionic Dyes from Wastewater with a Water‐stable and Eco‐friendly Fe‐MOF via Host‐guest Encapsulation,” Journal of Cleaner Production 239 (2019): 117767, 10.1016/j.jclepro.2019.117767.

[75] Z. Xu , J. Zhang , T. Pan , et al., “Encapsulation of Hydrophobic Guests within Metal–Organic Framework Capsules for Regulating Host–Guest Interaction,” Chemistry of Materials 32 (2020): 3553–3560, 10.1021/acs.chemmater.0c00684.

[76] T. Friščić , D. G. Reid , I. Halasz , R. S. Stein , R. E. Dinnebier , and M. J. Duer , “Ion‐and Liquid‐assisted Grinding: Improved Mechanochemical Synthesis of Metal–organic Frameworks Reveals Salt Inclusion and Anion Templating,” Angewandte Chemie International Edition 49 (2010): 712–715, 10.1002/anie.200906583. 20017178

[77] V. Neubertová , V. Švorčík , and Z. Kolská , “Amino‐modified ZIF‐8 for Enhanced CO_2_ Capture: Synthesis, Characterization and Performance Evaluation,” Microporous and Mesoporous Materials 366 (2024): 112956, 10.1016/j.micromeso.2023.112956.

[78] G. Orlandi and F. Negri , “Electronic States and Transitions in C_60_ and C_70_ Fullerenes,” Photochemical & Photobiological Sciences 1 (2002): 289–308, 10.1039/b200178k. 12653466

[79] J. Zhang , M. Gao , Y. Wang , et al., “Light‐induced Charge Transfer from a Fullerene to a Zeolitic Imidazolate Framework Enhances Alkaline Electrocatalytic Hydrogen Production,” Nanoscale 17 (2025): 2193–2199, 10.1039/D4NR04236K. 39655515

[80] A. T. A. Duong , H. V. Nguyen , M. V. Tran , et al., “Influence of ZIF‐9 and ZIF‐12 Structure on the Formation of a Series of New Co/N‐doped Porous Carbon Composites as Anode Electrodes for High‐performance Lithium‐ion Batteries,” RSC Advances 13 (2023): 17370–17383, 10.1039/D3RA02802J. 37304771 PMC10251121

[81] S. Chongdar , A. Ghosh , R. Bal , and A. Bhaumik , “Microwave‐assisted Synthesis of ZIF‐9@ X GO Composites as Cooperative Electrocatalysts for Electro‐oxidation of Benzyl Alcohols Coupled with H_2_ Production,” Journal of Materials Chemistry A 12 (2024): 233–246, 10.1039/D3TA04894B.

[82] S. Zhang , H. Dang , F. Rong , et al., “Multiple Cobalt Active Sites Evenly Embedded in Mesoporous Carbon Nanospheres Derived from a Polymer‐metal‐organic Framework: Efficient Removal and Photodegradation of Malachite Green,” RSC Advances 12 (2022): 32307–32317, 10.1039/D2RA04906F. 36425679 PMC9648500

[83] J. Zhang , W. Suo , Y. Han , et al., “Co Nanoparticles Encapsulated in N‐doped Carbon Nanotube Materials Derived from New Metal–organic Frameworks for Oxygen Electrocatalysis,” Journal of Materials Chemistry A 13 (2025): 669–679, 10.1039/D4TA07187E.

[84] P. Bhatia , R. Chandra , and M. Nath , “Controlled Synthesis of ZIF‐11 with Varied Particle Size: Effective Adsorbent for Industrial Pollutants and Host for Storage of Gaseous CO_2_, H_2_ and CH_4_ ,” Materials Chemistry and Physics 320 (2024): 129413, 10.1016/j.matchemphys.2024.129413.

[85] Y. Xie , W. Zhou , J.‐W. Yin , et al., “A Novel Electrochemical Sensor Based on La‐MOF@C60‐β‐cyclodextrin Composite for Sensitive Detection of Dichlorophen in Lake and Tap Water,” Journal of Environmental Chemical Engineering 13 (2025): 115388, 10.1016/j.jece.2025.115388.

[86] M. E. Potter , C. P. Ross , D. Gianolio , R. Rios , and R. Raja , “Cobalt‐containing Zeolitic Imidazole Frameworks for C–H Activation Using Visible‐light Redox Photocatalysis,” Catalysis Science & Technology 10 (2020): 7262–7269, 10.1039/D0CY01061H.

[87] H. S. Kim , “Enhancement of Photocatalytic Activity of Tris (bipyridine) Ruthenium by Encapsulation in Zeolitic Imidazolate Framework‐11 and 12,” Applied Science and Convergence Technology 30 (2021): 115–117, 10.5757/ASCT.2021.30.4.115.

[88] Y. Hidalgo‐Rosa , M. Saavedra‐Torres , B. D. Koivisto , et al., “Rare‐earth‐based Metal–organic Frameworks with Improved Visible‐light‐harvesting Properties: a Quantum Chemistry Study,” Journal of Materials Science 58 (2023): 8862–8877, 10.1007/s10853-023-08581-6.

[89] L. Jing , P. Li , Z. Li , D. Ma , and J. Hu , “Influence of π–π Interactions on Organic Photocatalytic Materials and Their Performance,” Chemical Society Reviews 54 (2025): 2054–2090, 10.1039/D4CS00029C. 39849932

[90] R. Anand , F. Borghi , F. Manoli , et al., “Host–Guest Interactions in Fe(III)‐Trimesate MOF Nanoparticles Loaded with Doxorubicin,” The Journal of Physical Chemistry B 118 (2014): 8532–8539, 10.1021/jp503809w. 24960194

[91] M. Souto , J. Calbo , S. Mañas‐Valero , A. Walsh , and G. M. Espallargas , “Charge‐transfer Interactions between Fullerenes and a Mesoporous Tetrathiafulvalene‐based Metal–organic Framework,” Beilstein Journal of Nanotechnology 10 (2019): 1883–1893, 10.3762/bjnano.10.183. 31598454 PMC6774073

[92] A. Arissa , T. Rose , N. Leick , S. Grimme , J. C. Johnson , and J. V. Lockard , “Charge Transfer and Recombination Pathways through Fullerene Guests in Porphyrin‐Based MOFs,” The Journal of Physical Chemistry C 129 (2025): 8215–8227, 10.1021/acs.jpcc.5c00161. PMC12333347 40786243

[93] A. S. Buchelnikov , V. V. Kostyukov , M. P. Yevstigneev , and Y. I. Prylutskyy , “Mechanism of Complexation of the Phenothiazine Dye Methylene Blue with Fullerene C_60_ ,” Russian Journal of Physical Chemistry A 87 (2013): 662–667, 10.1134/S0036024413040067.

[94] A. Das and M. K. Adak , “Photo‐catalyst for Wastewater Treatment: A Review of Modified Fenton, and Their Reaction Kinetics,” Applied Surface Science Advances 11 (2022): 100282, 10.1016/j.apsadv.2022.100282.

[95] Q. Cai , Z. Hu , Q. Zhang , B. Li , and Z. Shen , “Fullerene (C_60_)/CdS Nanocomposite with Enhanced Photocatalytic Activity and Stability,” Applied Surface Science 403 (2017): 151–158, 10.1016/j.apsusc.2017.01.135.

[96] J. Hu , P. Zhang , W. An , L. Liu , Y. Liang , and W. Cui , “In‐situ Fe‐doped G‐C_3_N_4_ Heterogeneous Catalyst via Photocatalysis‐Fenton Reaction with Enriched Photocatalytic Performance for Removal of Complex Wastewater,” Applied Catalysis B: Environmental 245 (2019): 130–142, 10.1016/j.apcatb.2018.12.029.

[97] X. Wang , X. Zhang , Y. Zhang , et al., “Nanostructured Semiconductor Supported Iron Catalysts for Heterogeneous Photo‐Fenton Oxidation: A Review,” Journal of Materials Chemistry A 8 (2020): 15513–15546, 10.1039/D0TA04541A.

[98] Z. Zhu , L. Wang , C. Wang , and J. Zhao , “Coupled Nanoconfinement Effect and Host–Guest Interaction in the Fe(III)‐Oxalate/LDH System for a Highly Efficient Photoassisted Fenton Reaction,” ACS ES&T Engineering 5 (2025): 2007–2018, 10.1021/acsestengg.5c00135.

[99] M. Zhang , X. Liang , Y. Gao , and Y. Liu , “C60‐ and CdS‐Co‐Modified Nano‐Titanium Dioxide for Highly Efficient Photocatalysis and Hydrogen Production,” Materials 17 (2024): 1206, 10.3390/ma17051206. 38473677 PMC10934443

[100] F. Audino , L. O. Conte , A. V. Schenone , M. Pérez‐Moya , M. Graells , and O. M. Alfano , “A Kinetic Study for the Fenton and Photo‐Fenton Paracetamol Degradation in an Annular Photoreactor,” Environmental Science and Pollution Research 26 (2019): 4312–4323, 10.1007/s11356-018-3098-4. 30229488 PMC8298369

[101] J. He , Y. Zhang , X. Zhang , and Y. Huang , “Highly Efficient Fenton and Enzyme‐mimetic Activities of NH2‐MIL‐88B(Fe) Metal Organic Framework for Methylene Blue Degradation,” Scientific Reports 8 (2018): 5159, 10.1038/s41598-018-23557-2. 29581533 PMC5980107

[102] E. Haque , J. W. Jun , and S. H. Jhung , “Adsorptive Removal of Methyl Orange and Methylene Blue from Aqueous Solution with a Metal‐organic Framework Material, Iron Terephthalate (MOF‐235),” Journal of Hazardous Materials 185 (2011): 507–511, 10.1016/j.jhazmat.2010.09.035. 20933323

[103] I. A. Lázaro , H. Szalad , P. Valiente , J. Albero , H. García , and C. Martí‐Gastaldo , “Tuning the Photocatalytic Activity of Ti‐Based Metal–Organic Frameworks through Modulator Defect‐Engineered Functionalization,” ACS Applied Materials & Interfaces 14 (2022): 21007–21017, 10.1021/acsami.2c02668. 35482456 PMC9100481

[104] A. Malik and M. Nath , “Multicore‒Shell Nanocomposite Formed by Encapsulation of WO3 in Zeolitic Imidazolate Framework (ZIF‐8): As an Efficient Photocatalyst,” Journal of Environmental Chemical Engineering 7 (2019): 103401, 10.1016/j.jece.2019.103401.

[105] S. Payra , S. Challagulla , Y. Bobde , C. Chakraborty , B. Ghosh , and S. Roy , “Probing the Photo‐ and Electro‐catalytic Degradation Mechanism of Methylene Blue Dye over ZIF‐derived ZnO,” Journal of Hazardous Materials 373 (2019): 377–388, 10.1016/j.jhazmat.2019.03.053. 30933860

[106] J.‐L. Qiu , J. Su , N. Muhammad , et al., “Facile Encapsulating Ag Nanoparticles into a Tetrathiafulvalene‐based Zr‐MOF for Enhanced Photocatalysis,” Chemical Engineering Journal 427 (2022): 131970, 10.1016/j.cej.2021.131970.

[107] L. Cen , T. Tang , F. Yu , et al., “Fabrication of ZIF‐8/TiO_2_ Electrospinning Nanofibers for Synergistic Photodegradation in Dyeing Wastewater,” Journal of Industrial and Engineering Chemistry 126 (2023): 537–545, 10.1016/j.jiec.2023.06.042.

[108] S. Li , S. Sun , H. Wu , C. Wei , and Y. Hu , “Effects of Electron‐donating Groups on the Photocatalytic Reaction of MOFs,” Catalysis Science & Technology 8 (2018): 1696–1703, 10.1039/C7CY02622F.

[109] S. M. Pratik , L. Gagliardi , and C. J. Cramer , “Boosting Photoelectric Conductivity in Porphyrin‐Based MOFs Incorporating C_60_ ,” The Journal of Physical Chemistry C 124 (2020): 1878–1887, 10.1021/acs.jpcc.9b10834.

[110] B. Kaur , V. Soni , R. Kumar , et al., “Recent Advances in Manipulating Strategies of NH_2_‐functionalized Metallic Organic Frameworks‐based Heterojunction Photocatalysts for the Sustainable Mitigation of Various Pollutants,” Environmental Research 259 (2024): 119575, 10.1016/j.envres.2024.119575. 38986799

[111] A. V. Desai , S. M. Vornholt , L. L. Major , et al., “Surface‐Functionalized Metal–Organic Frameworks for Binding Coronavirus Proteins,” ACS Applied Materials & Interfaces 15 (2023): 9058–9065, 10.1021/acsami.2c21187. 36786318 PMC9940617

[112] A. Habibi‐Yangjeh , S. Asadzadeh‐Khaneghah , S. Feizpoor , and A. Rouhi , “Review on Heterogeneous Photocatalytic Disinfection of Waterborne, Airborne, and Foodborne Viruses: Can We Win against Pathogenic Viruses?,” Journal of Colloid and Interface Science 580 (2020): 503–514, 10.1016/j.jcis.2020.07.047. 32711201 PMC7361121

[113] X. Zhang , Y. Liu , and J. Yuan , “Amino‐functionalized Fe/Co Bimetallic MOFs for Accelerated Fe (III)/Fe (II) Cycling and Efficient Degradation of Sulfamethoxazole in Fenton‐Like System,” Frontiers in Chemistry 13 (2025): 1579108, 10.3389/fchem.2025.1579108. 40224220 PMC11986425

[114] Y. Yamaguchi , S. Usuki , Y. Kanai , et al., “Selective Inactivation of Bacteriophage in the Presence of Bacteria by Use of Ground Rh‐Doped SrTiO_3_ Photocatalyst and Visible Light,” ACS Applied Materials & Interfaces 9 (2017): 31393–31400, 10.1021/acsami.7b07786. 28872820

[115] M. Cho , H. Chung , W. Choi , and J. Yoon , “Different Inactivation Behaviors of MS‐2 Phage and Escherichia coli in TiO_2_ Photocatalytic Disinfection,” Applied and Environmental Microbiology 71 (2005): 270–275, 10.1128/AEM.71.1.270-275.2005. 15640197 PMC544209

[116] B. Ran , L. Ran , Z. Wang , et al., “Photocatalytic Antimicrobials: Principles, Design Strategies, and Applications,” Chemical Reviews 123 (2023): 12371–12430, 10.1021/acs.chemrev.3c00326. 37615679

[117] S. S. Ndzimandze , O. T. Mahlangu , A. A. Muleja , A. T. Kuvarega , and T. T. I. Nkambule , “Toward the Development and Optimization of a Solar Photo‐Fenton Degradation Method for Natural Organic Matter Removal in Selected South African Drinking Water Treatment Plants,” ACS Omega 10 (2025): 28752–28762, 10.1021/acsomega.4c10339. 40686964 PMC12268384

[118] Y. Shen , J. Kang , L. Guo , F. Qiu , Y. Fan , and S. Zhang , “Recent Progress on the Application of MOFs and Their Derivatives in Adsorbing Emerging Contaminants,” Separation and Purification Technology 350 (2024): 127955, 10.1016/j.seppur.2024.127955.

[119] M. Moradi , R. R. Kalantary , A. Esrafili , A. J. Jafari , and M. Gholami , “Visible Light Photocatalytic Inactivation of Escherichia coli by Natural Pyrite Assisted by Oxalate at Neutral pH,” Journal of Molecular Liquids 248 (2017): 880–889, 10.1016/j.molliq.2017.10.115.

[120] S. A. Abd Elmohsen , G. E. Daigham , S. A. Mohmed , and N. M. Sidkey , “Photocatalytic degradation of biological contaminant (E. coli) in drinking water under direct natural sunlight irradiation using incorporation of green synthesized TiO_2_, Fe_2_O_3_ nanoparticles,” Biomass Conversion and Biorefinery 15 (2025): 6713–6734.

[121] C. P. Rodrigues , R. L. Ziolli , and J. R. Guimarães , “Inactivation of Escherichia coli in Water by TiO_2_‐assisted Disinfection Using Solar Light,” Journal of the Brazilian Chemical Society 18 (2007): 126–134, 10.1590/S0103-50532007000100014.

[122] G. Li , X. Nie , J. Chen , et al., “Enhanced Visible‐light‐driven Photocatalytic Inactivation of Escherichia coli Using G‐C3N4/TiO2 Hybrid Photocatalyst Synthesized Using a Hydrothermal‐calcination Approach,” Water Research 86 (2015): 17–24, 10.1016/j.watres.2015.05.053. 26084941

[123] S. Sontakke , C. Mohan , J. Modak , and G. Madras , “Visible Light Photocatalytic Inactivation of Escherichia coli with Combustion Synthesized TiO2,” Chemical Engineering Journal 189‐190 (2012): 101–107, 10.1016/j.cej.2012.02.036.

[124] A.‐G. Rincón and C. Pulgarin , “Bactericidal Action of Illuminated TiO_2_ on Pure Escherichia coli and Natural Bacterial Consortia: Post‐irradiation Events in the Dark and Assessment of the Effective Disinfection Time,” Applied Catalysis B: Environmental 49 (2004): 99–112, 10.1016/j.apcatb.2003.11.013.

[125] R. Cheng , M. Kang , Z. Shen , L. Shi , and X. Zheng , “Visible‐light‐driven Photocatalytic Inactivation of Bacteriophage f2 by Cu‐TiO_2_ Nanofibers in the Presence of Humic Acid,” Journal of Environmental Sciences 77 (2019): 383–391, 10.1016/j.jes.2018.09.017. 30573103

[126] D. A. Pino‐Sandoval , M. E. Cantú‐Cárdenas , V. Rodríguez‐González , et al., “Solar Heterogeneous Photo‐Fenton for Complete Inactivation of Escherichia coli and Salmonella Typhimurium in Secondary‐treated Wastewater Effluent,” Chemosphere 342 (2023): 140132, 10.1016/j.chemosphere.2023.140132. 37690560

[127] P. Prieto‐Laria , P. Fernández‐Ibáñez , A. R. Ruiz‐Salvador , et al., “Cu or Fe‐Exchanged Natural Clinoptilolite as Sustainable Light‐Assisted Catalyst for Water Disinfection at Near Neutral pH,” ChemPlusChem 90 (2025): 202500225, 10.1002/cplu.202500225. PMC12605726 40964973

[128] P. Prieto‐Laria , A. Jiménez‐Rodríguez , A. R. Ruiz‐Salvador , et al., “From the Lab to the River: Bimetallic Clinoptilolite Photocatalyst for Antibiotic‐Resistant Bacteria and Emerging Contaminants Removal,” Journal of Environmental Chemical Engineering 13 (2025): 116663, 10.1016/j.jece.2025.116663.

[129] N. Rodríguez‐Sánchez , B. Bhattacharya , F. Emmerling , et al., “Engineering a Multivariate Cobalt Metal–Organic Framework for High Photocatalytic Activity: The Impact of Mixed Ligands and Metal Incorporation in a Visible Light‐Driven Heterogeneous Photo‐Fenton Reaction for Water Treatment,” Nanoscale Advances 7 (2025): 2255–2265, 10.1039/D4NA00954A. 40028495 PMC11868913

