# Supplementary material for: Mechanochemical Fullerene Nanoencapsulation in Amino‐Functionalized ZIF‐12 for Visible‐Light Disinfection of Waterborne Viruses and Bacteria
Source: Small. 2026 Feb 23;22(23):e12881. doi: 10.1002/smll.202512881 (PMC13100564; doi:10.1002/smll.202512881)
Supplement: Supplementary file 1 — Supporting File: smll72928‐sup‐0001‐SuppMat.docx. [file SMLL-22-e12881-s001.docx]

Supporting information

**Mechanochemical Fullerene Nanoencapsulation in Amino-Functionalized ZIF-12 for Visible-Light Disinfection of Waterborne Viruses and Bacteria**

*Noelia Rodríguez-Sánchez, Menta Ballesteros, Carsten Prinz, Inés Canosa, Amando Flores Díaz, Biswajit Bhattacharya^*^, A. Rabdel Ruiz-Salvador*, Franziska Emmerling^*^*

N. Rodríguez-Sánchez, C. Prinz, F. Emmerling, B. Bhattacharya

Structure Analysis department, BAM Federal Institute for Materials Research and Testing, Berlin, Germany

E-mail: [biswajit.bhattacharya@bam.de](mailto:biswajit.bhattacharya@bam.de), franziska.emmerling@bam.de

M. Ballesteros,

Department of Molecular Biology and Biochemistry Engineering, Universidad Pablo de Olavide, Seville, Spain.

I. Canosa, A. Flores-Díaz

Centro Andaluz de Biología del Desarrollo/Universidad Pablo de Olavide/ Junta de Andalucía, Seville, Spain

A. R. Ruiz-Salvador

Department of Physical, Chemical and Natural Systems, Universidad Pablo de Olavide, Seville, Spain.

E-mail: [rruisal@upo.es](mailto:rruisal@upo.es)

**Adsorption kinetic study**

Pseudo-first-order model, pseudo-second-order model and Intraparticle diffusion model or Weber-Morris model adsorption kinetics were analyzed to investigate the adsorption behavior of the contaminant onto the synthesized material and to determine the rate-controlling mechanisms during the dark period prior to photocatalysis and photo-Fenton assays. A known amount of the photocatalyst (0.025 g/L) was dispersed in the contaminant solution with a fixed initial concentration (0.05 g/L), and samples were taken every 15 minutes. The residual contaminant concentration was analyzed, and the adsorption capacity at time t (q_t_, mg g⁻¹) was calculated according to **Equation 1**:^1^

$q_{t}= \frac{\left( C_{0}-C_{t} \right)\times V}{m}$ **Equation 1**

where C_0_​ and C_t_​ (mg L⁻¹) are the contaminant concentrations at initial time and at time t, respectively, V (L) is the solution volume, and m (g) is the mass of adsorbent. The suitability of the kinetic models was evaluated by comparing the correlation coefficients (R^2^) and the agreement between experimental and calculated adsorption capacities.

**Pseudo-first-order kinetic model (PFO)**

The pseudo-first-order kinetic model was applied to describe the adsorption rate based on the assumption that the rate of occupation of adsorption sites is proportional to the number of unoccupied sites. The linear form of the PFO model is expressed as **Equation 2**:^1,2^

$\ln\left( q_{e}-q_{t} \right)=lnq_{e}-k_{1}t$ **Equation 2**

where q_e_​ (mg g⁻¹) and qt (mg g⁻¹) are the adsorption capacities at equilibrium and at time t, respectively, and k_1_​ (min⁻¹) is the pseudo-first-order rate constant. The values of k_1_ and q_e_ were obtained from the slope and intercept of the linear plot of ln (q_e_-q_t_) versus t.

**Pseudo-second-order kinetic model (PSO)**

The pseudo-second-order kinetic model assumes that the adsorption process is controlled by chemisorption involving valence forces or electron sharing between adsorbent and adsorbate. The linearized form of the PSO model is given by **Equation 3**:^1,3^

$\frac{t}{q_{t}}= \frac{1}{k_{2}q_{e}^{2}}+ \frac{t}{q_{e}}$ **Equation 3**

where k_2_​ (g mg⁻¹ min⁻¹) is the pseudo-second-order rate constant. The parameters q_e_ and k_2_​ were calculated from the slope and intercept of the linear plot of t/q_t_​ versus t.

**Intraparticle diffusion model (Weber-Morris)**

To investigate the contribution of intraparticle diffusion to the adsorption mechanism, the Weber-Morris intraparticle diffusion model was employed. This model is described by **Equation 4**:^1,4^

$q_{t}= k_{\mathrm{id}}t^{\frac{1}{2}}+C$ **Equation 4**

where k_id​_ (mg g⁻¹ min⁻¹/²) is the intraparticle diffusion rate constant and C (mg g⁻¹) is the intercept related to the boundary layer thickness. If the plot of qt​ versus t^1/2^ is linear and passes through the origin, intraparticle diffusion is the sole rate-controlling step; otherwise, multiple adsorption mechanisms are involved.

REFERENCES

1 K. Jedynak, M. Repelewicz, K. Kurdziel and D. Wideł, *Desalination and Water Treatment*, 2021, **220**, 363–379.

2 X. Guo and J. Wang, *Journal of Molecular Liquids*, 2019, **288**, 111100.

3 Y. S. Ho and G. McKay, *Process Biochemistry*, 1999, **34**, 451–465.

4 W. J. Weber and J. C. Morris, *J. Sanit. Engrg. Div.*, 1963, **89**, 31–59.

**Degradation kinetic study**

Photocatalysis and photo-Fenton process was analyzed through different kinetic models. Primarily, first-order kinetic model of Langmuir-Hinshelwood (L-H) was analyzed following **Equation 5** where r is the reaction rate, k_cat_ is the catalytic rate constant, K is the adsorption equilibrium constant, and C is the concentration of the reactant. 1+ K⋅ C ≈ 1 was assumed due to the low concentration of MB (5 mg/l), being for instance the reaction rate represented in **Equation 6**, k_app_ represent the apparent first-order rate constant. Integration of Equation 2 lead to a linear relationship between the natural logarithm of concentration and time allowing for a simpler mathematical treatment in **Equation 7**. Where [c] is concentration of each point, [c_0_] is the initial concentration, *k* is the velocity constant and t is the time.

$r=-\frac{dC}{dt}=\frac{k_{cat}\cdot K\cdot C}{1+K\cdot C}$ **Equation 5**

$r \approx k_{cat} \cdot K\cdot C= k_{app}\cdot C$ **Equation 6**

$-ln\frac{\left[ c \right]}{\left[ c \right]_{0}}= k_{1} t$  **Equation 7**

zero-order and pseudo-second-order kinetic models were also evaluated in order to find the better fit of the kinetic model of the degradation (**Equation 8 and 9**)

$C_{0}- C_{t}= K_{o} \times t$ **Equation 8**

$\frac{1}{C_{t}}= k_{2} \times t$ **Equation 9**

**Band Gap Estimation by Tauc Plot Analysis of DRS Data**

The optical band gap of the samples was estimated from UV–Vis diffuse reflectance spectroscopy (DRS) data by applying the Tauc formalism. First, the diffuse reflectance spectra were converted to the Kubelka–Munk function, *F(R)*, according to:

$$F \left( R \right)= \frac{(1-R)^{2}}{2R}$$

where *R* is the reflectance. The optical absorption coefficient (α) is proportional to *F(R)*, and the band gap can be determined by plotting (*F(R)*⋅*hν*)^n^ against the photon energy (*hν*), where n depends on the nature of the electronic transition: n=2 for direct allowed transitions and n=1/2 for indirect allowed transitions. The linear region of the resulting Tauc plot is extrapolated towards the energy axis, and the intercept with the abscissa gives the estimated band gap energy (*Eg*). This approach allows the assessment of the optical properties of powdered photocatalysts and is widely used for MOFs and other porous semiconductors.





**Figure S.1.** FT-IR spectra of C_60_@ZIF-12 with different loading of C_60_, fullerene and ZIF-12.

**Table S.1.** Specific surface area (BET) analysis of ZIF-12, ZIF-12-NH_2_, C_60_@ZIF-12 and C_60_@ZIF-12-NH_2_

| **Material** | **specific surface area (BET)  [****m²/g]** | **BET uncertainty [m²/g]** | **C** | **Cor. Coeff.** |
| --- | --- | --- | --- | --- |
| ZIF-12 | 391.77 | 1.34 | 741.38 | 0.9999 |
| ZIF-12-NH_2_ | 200.20 | 0.48 | 619.73 | 0.9999 |
| C_60_@ZIF-12 | 1.29 | 0.0055 | 13.24 | 0.9999 |
| C_60_@ZIF-12- NH_2_ | 2.55 | 0.04 | 16.15 | 0.9999 |


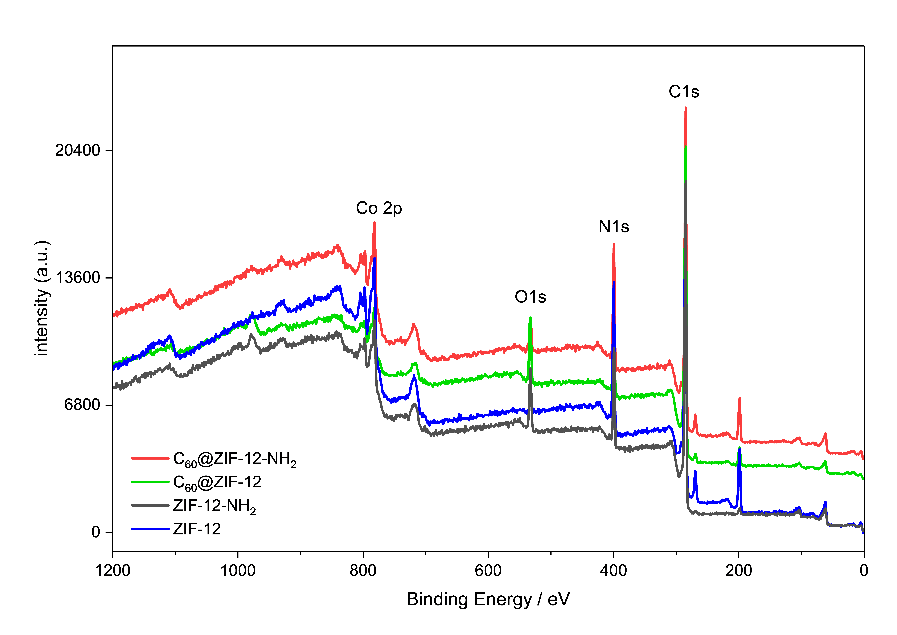


**Figure S.2**. XPS summary data of N, C, O and Co atoms for ZIF-12, ZIF-12-NH_2_, C_60_@ZIF-12 and C_60_@ZIF-12-NH_2_


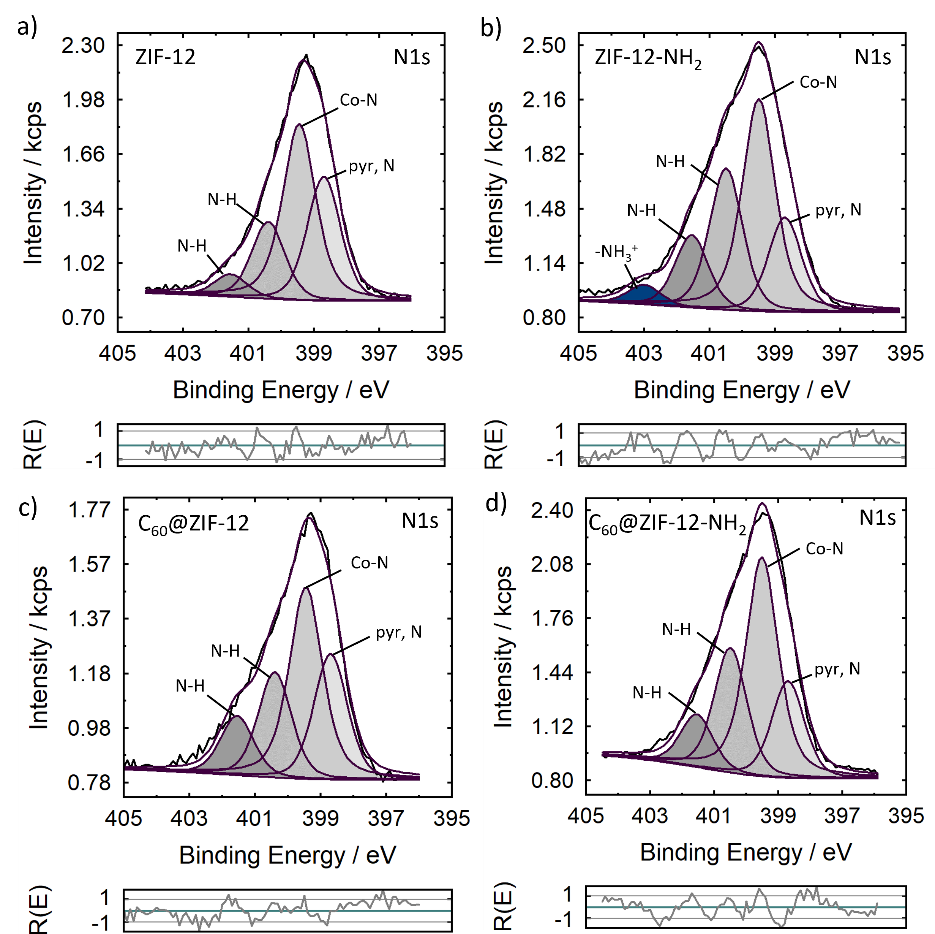


**Figure S.3.** High resolution XPS data of N1s region for ZIF-12 a), ZIF-12-NH_2_ b), C_60_@ZIF-12 c) and C_60_@ZIF-12-NH_2_ d)


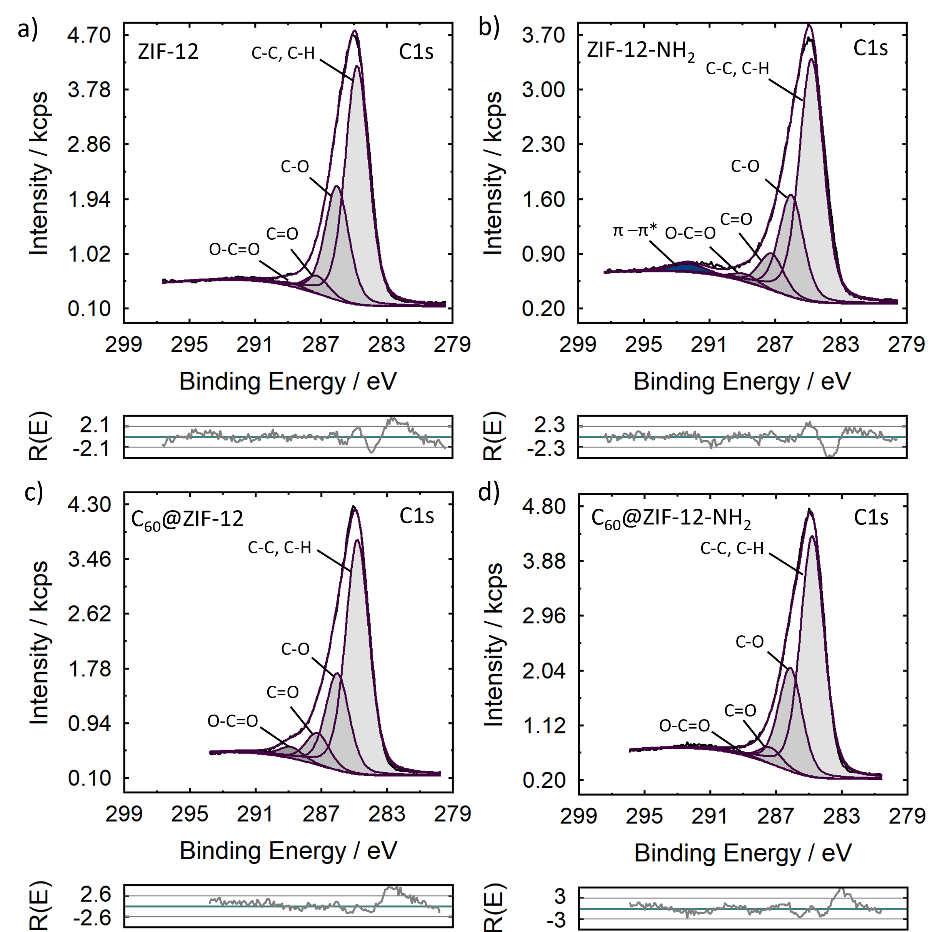


**Figure S.4.** High resolution XPS data of C1s region for ZIF-12 a), ZIF-12-NH_2_ b), C_60_@ZIF-12 c) and C_60_@ZIF-12-NH_2_ d)


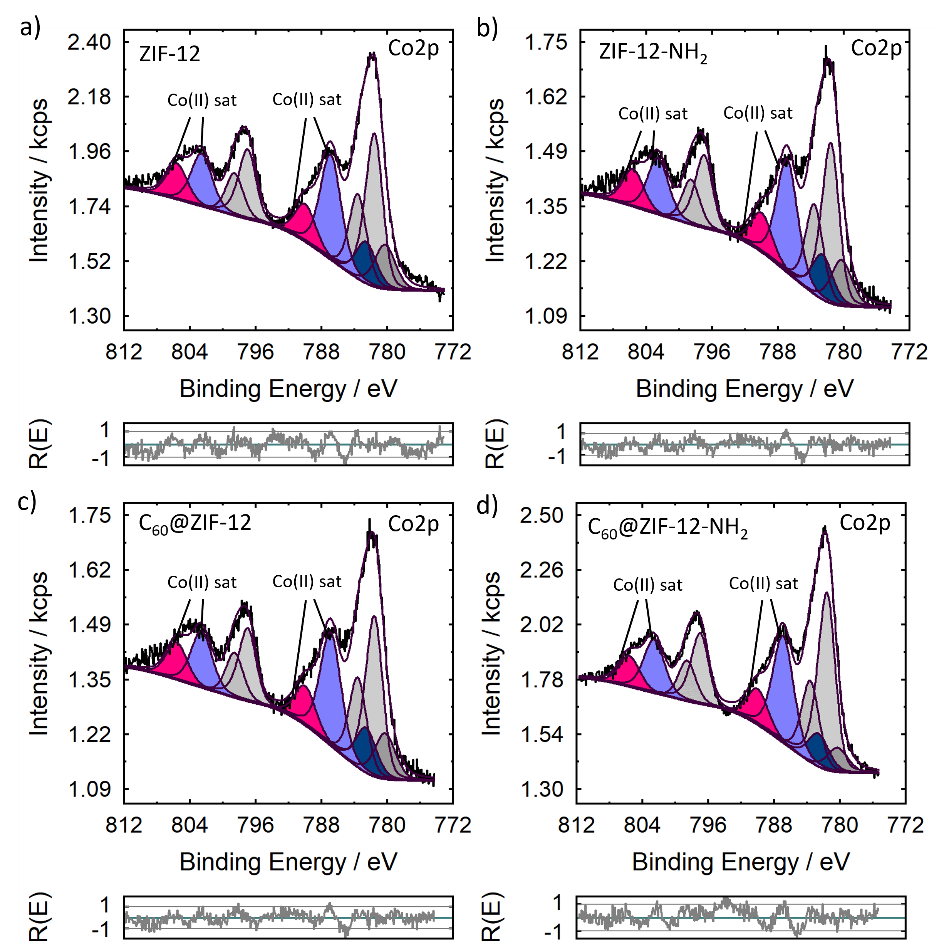


**Figure S.5.** High resolution XPS data of Co2p region for ZIF-12 a), ZIF-12-NH_2_ b), C_60_@ZIF-12 c) and C_60_@ZIF-12-NH_2_ d)


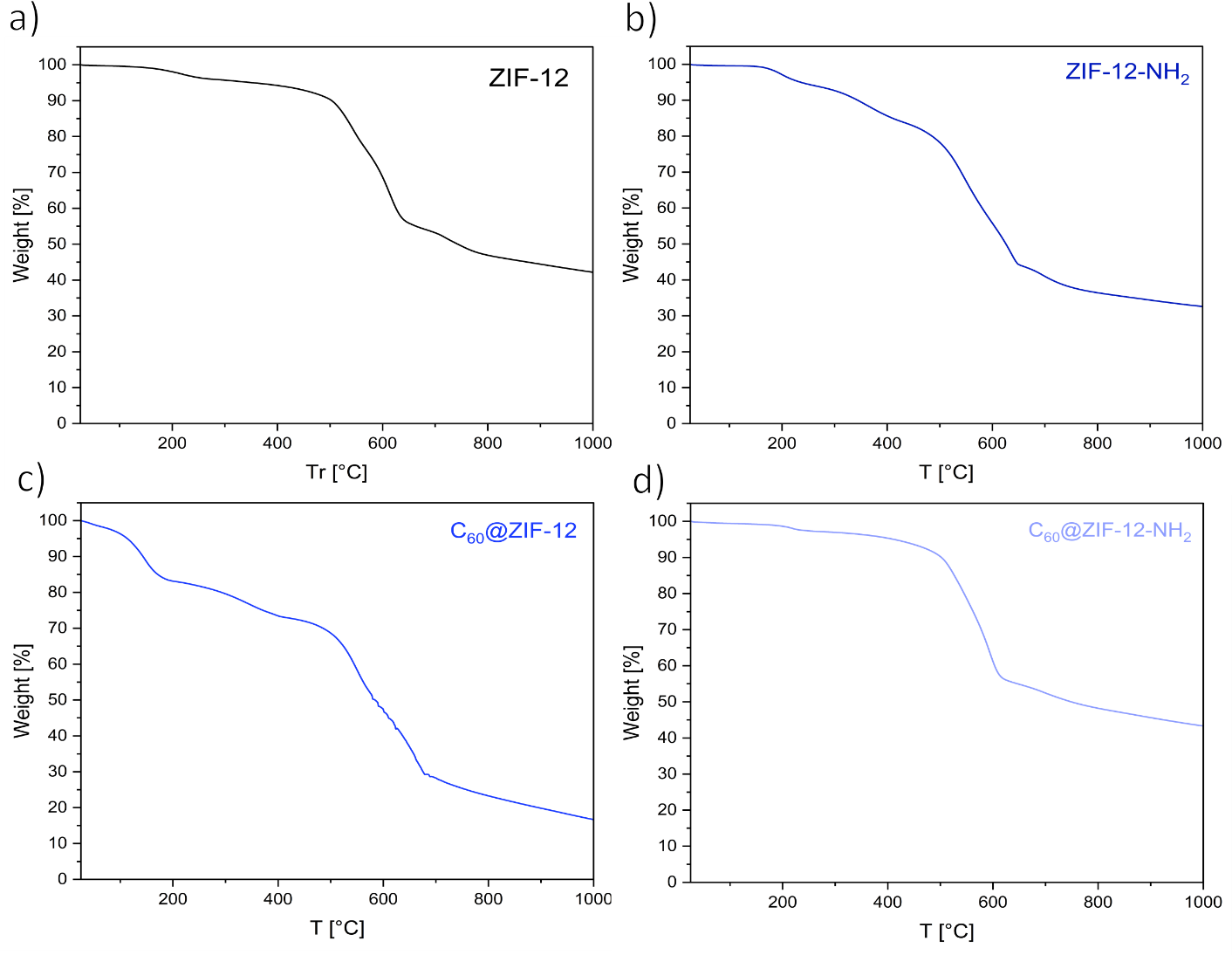


**Figure S.6.** Thermogravimetric analysis of ZIF-12 a), ZIF-12-NH₂ b), C_60_@ZIF-12 c) and C_60_@ZIF-12-NH₂ d)


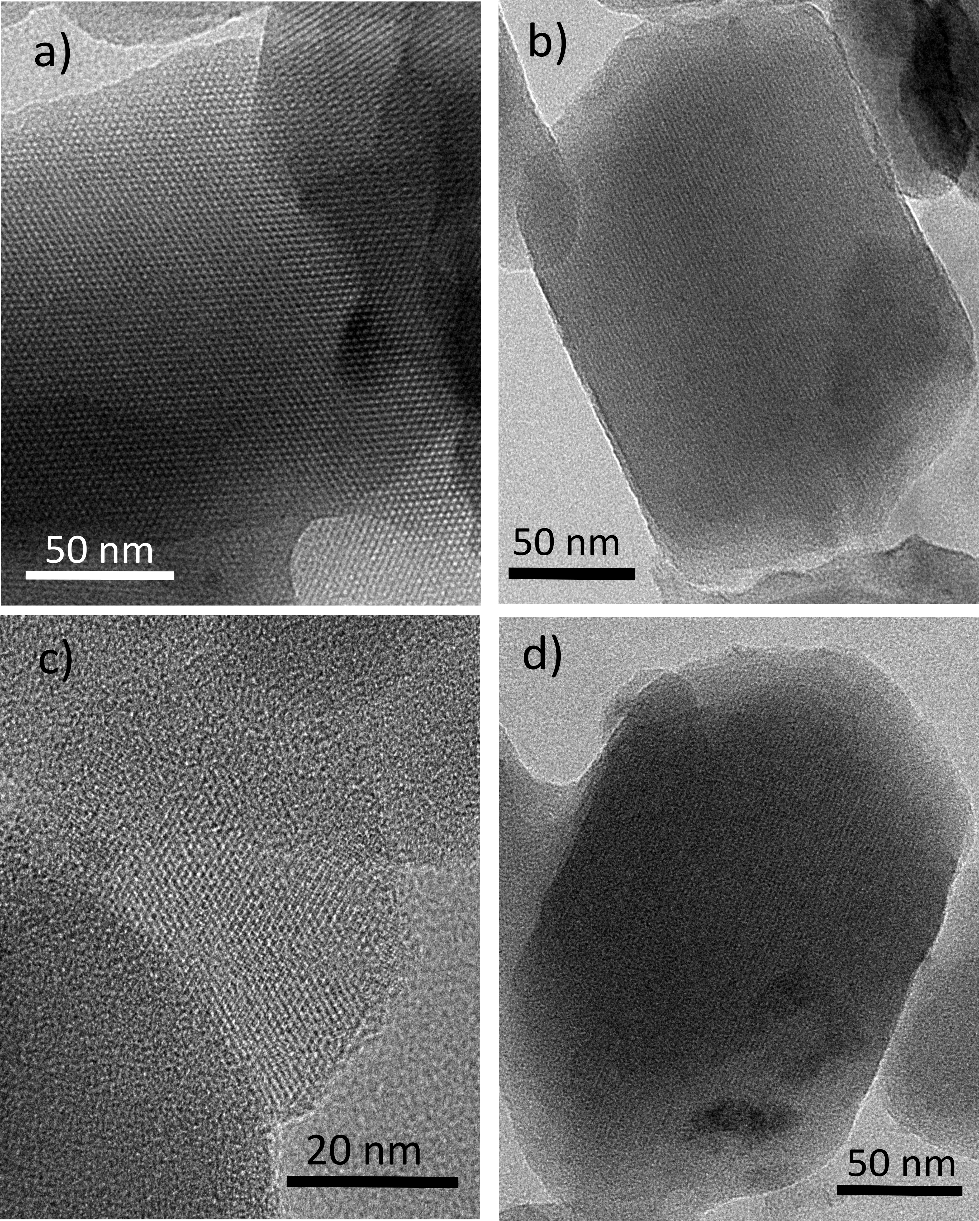


**Figure S.7.** HRTEM images of ZIF-12 a), ZIF-12-NH_2_ b), C_60_@ZIF-12 c) and C_60_@ZIF-12-NH_2_ d)

**Table S.2.** Degradation kinetics of MB using C_60_@ZIF-12 and different C_60_ loading by heterogeneous photo-Fenton.

| C_60_ loading | K | R^2^ | Kinetic model |
| --- | --- | --- | --- |
| 6 mg | K (Lmg^-1^min^-1^)  40.624 | 0.9996 | Pseudo-second order |
| 9 mg | K (min^-1^)  0.4244 | 0.9994 | First order |
| 12 mg | K (min^-1^)  0.4645 | 0.9757 | First order |
| 15 mg | K (min^-1^)  0.5 | 0.9883 | First order |

**Table S.3.** Degradation kinetics of MB using C_60_@ZIF-12-NH_2_ and different C_60_ loading by heterogeneous photo-Fenton.

| C_60_ loading | K (min^-1^) | R^2^ | Kinetic model |
| --- | --- | --- | --- |
| 9 mg | K (min^-1^)  0.6015 | 0.9989 | First order |
| 12 mg | K (min^-1^)  0.6199 | 0.9874 | First order |
| 15 mg | K (Lmg^-1^min^-1^)  126.78 | 0.9853 | Pseudo-second order |


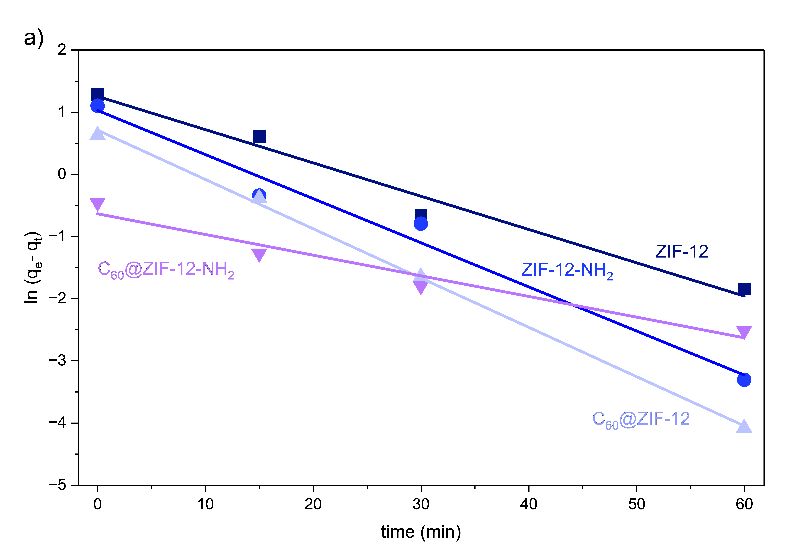

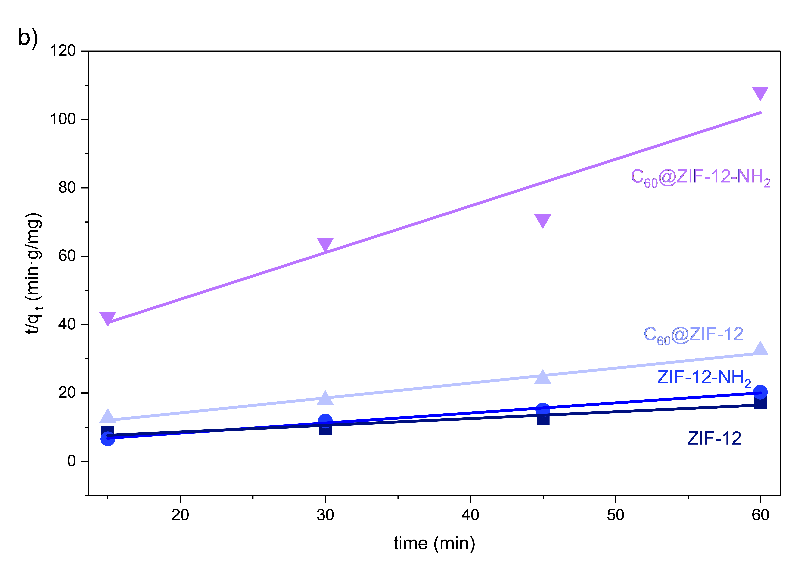
**
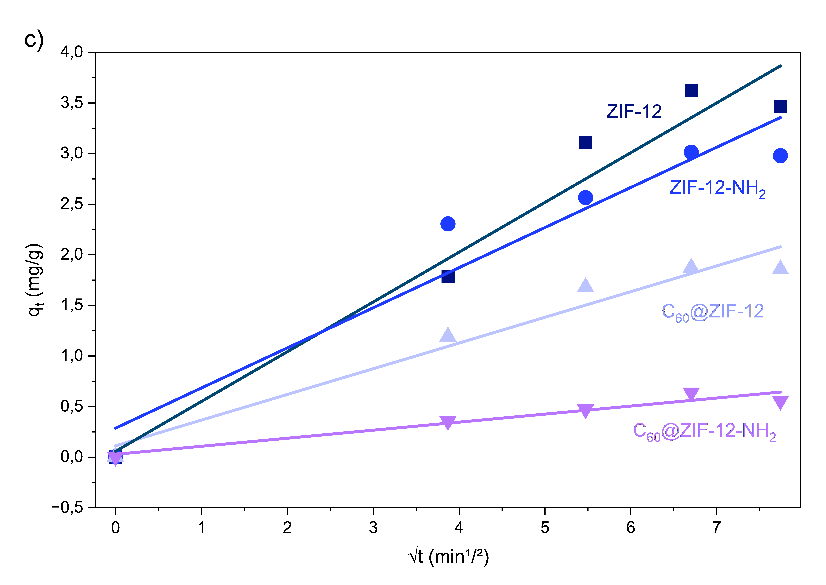
**

**Figure S.8**. adsorption kinetic analysis of pseudo first-order kinetic a) pseudo-second order kinetic b) and Weber-Morris kinetic c) for ZIF-12, ZIF-12-NH_2_, C_60_@ZIF-12 and C_60_@ZIF-12-NH_2_

**Table S.4.** Kinetic parameters for MB adsorption on modified ZIF-12 materials in darkness.

| MOF | k₁ (min⁻¹) | R²(PPO) | qₑ(PSO) (mg/g) | R²(PSO) | k_id | R²(Weber) |
| --- | --- | --- | --- | --- | --- | --- |
| ZIF-12 | 0.0536 | 0.9771 | 50.8 | 0.9292 | 0.492 | 0.9577 |
| ZIF-12-NH₂ | 0.0710 | 0.9804 | 3.40 | 0.9920 | 0.3966 | 0.9240 |
| C_60_@ZIF-12 | 0.0790 | 0.9986 | 2.29 | 0.9889 | 0.2541 | 0.9572 |
| C_60_@ZIF-12-NH₂ | 0.0333 | 0.9593 | 0.73 | 0.9279 | 0.0795 | 0.9408 |

*PPO: pseudo-first order; PSO: pseudo-second order; k_id: intraparticle diffusion constant (mg g⁻¹ min⁻¹/²)*

**Table S.5.** Degradation kinetics of MB using ZIF-12, ZIF-12-NH_2_, C_60_@ZIF-12 and C_60_@ZIF-12-NH_2_ by photocatalysis

|  | K (min^-1^) | R^2^ | Kinetic model |
| --- | --- | --- | --- |
| ZIF-12 | K (Lmg^-1^min^-1^)  0.8738 | 0.9839 | Pseudo-second order |
| ZIF-12-NH_2_ | K (min^-1^)  0.0038 | 0.9916 | First order |
| C_60_@ZIF-12 | K (min^-1^)  0.0049 | 0.9948 | First order |
| C_60_@ZIF-12-NH_2_ | K (min^-1^)  0.0076 | 0.9987 | First order |

**Table S.6.** Degradation kinetics of MB using ZIF-12, ZIF-12-NH_2_, C_60_@ZIF-12 and C_60_@ZIF-12-NH_2_ by heterogeneous photo-Fenton.

|  | K | R^2^ | Kinetic model |
| --- | --- | --- | --- |
| ZIF-12 | K (min^-1^)  0.001 | 0.9999 | First order |
| ZIF-12-NH_2_ | K (Lmg^-1^min^-1^)  188.31 | 0.9726 | Pseudo-second order |
| C_60_@ZIF-12 | K (min^-1^)  0.4645 | 0.9757 | First order |
| C_60_@ZIF-12-NH_2_ | K (min^-1^)  0.6199 | 0.9874 | First order |

**Table S.7.** Physicochemical parameters for river water samples. Total solids (TS); volaltile solids (VS); mineral solids (MS); total suspended solids (TSS); soluble chemical  oxygen demand (sCOD) and chemical oxygen demand (COD)

| Parameter | Value |  |
| --- | --- | --- |
| pH | 7.19 |  |
| O2 (mg/L) | 0.93 |  |
| T °C | 25.9 |  |
| Conductivity (µS/cm) | 1895 |  |
| Redox (mV) | -21.8 |  |
| Turbidity (NTU) | 81.23 |  |
| TS (mg/L) | 1291 | 25% VS; 75% MS |
| TSS /mg/L) | 80 | 56% VS; 44% MS |
| sCOD (mg/L) | 45 |  |
| COD (mg/L) | 101 |  |

**Table S.8.** STEM data of C_60_@ZIF-12-NH₂ before and after cycling.

|  | Element | Atomic Fraction (%) | Atomic Error (%) | Mass Fraction (%) | Mass Error |
| --- | --- | --- | --- | --- | --- |
| Before | C | 73.45 | 3.09 | 56.94 | 2.90 |
|  | N | 19.99 | 3.26 | 18.07 | 3.06 |
|  | Co | 6.57 | 0.87 | 24.98 | 2.69 |
| After | C | 73.46 | 3.14 | 58.15 | 2.90 |
|  | N | 20.69 | 3.32 | 19.10 | 3.17 |
|  | Co | 5.86 | 0.78 | 22.75 | 2.51 |


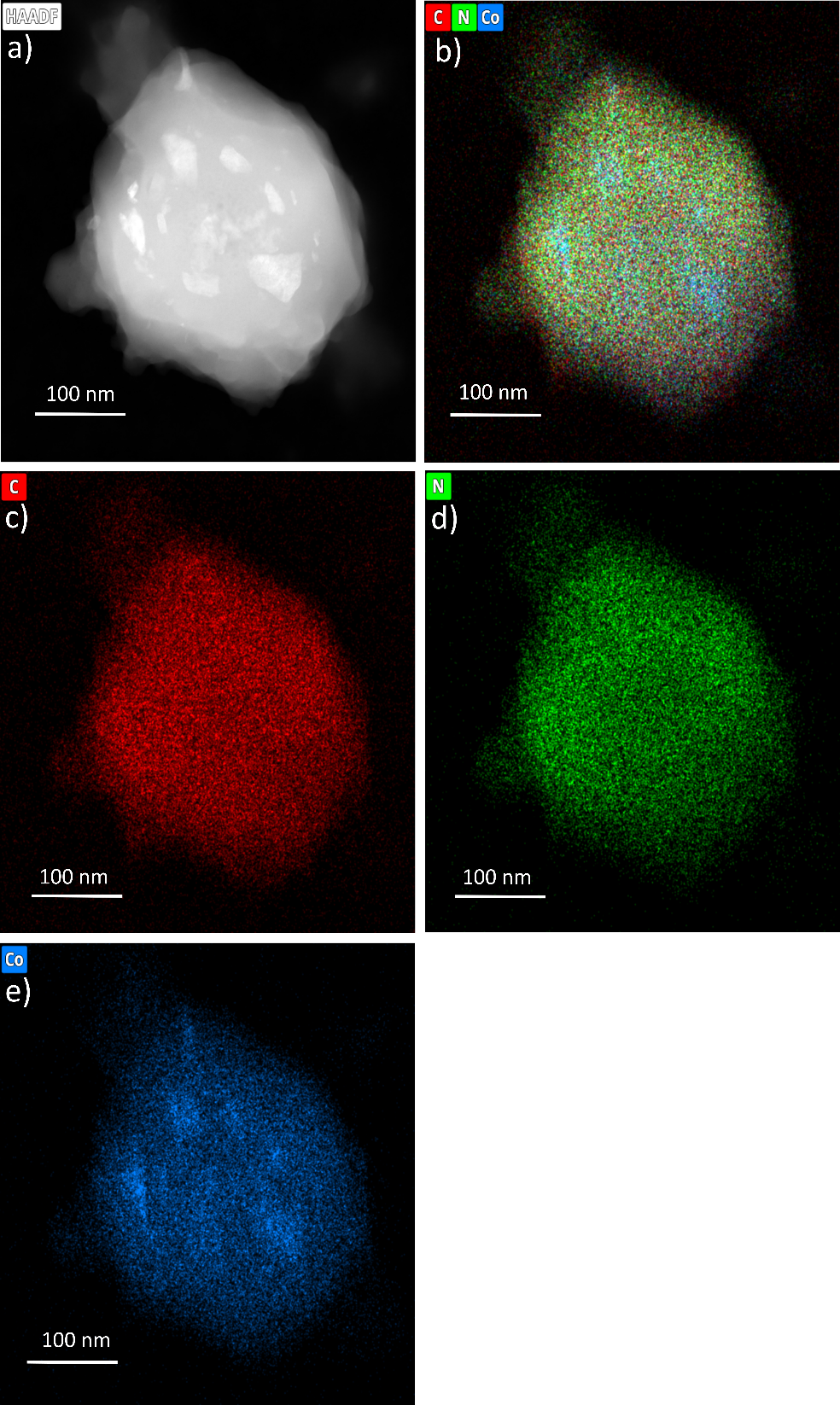


**Figure S.9.** STEM images of C_60_@ZIF-12-NH₂ after cycling where a) is the HAADF image, b) represent C,N,and Co atoms, c) carbon atoms, d) Nitrogen atoms and d) Cobalt atoms.
